# Defining the Genome Features of *Escherichia albertii*, an Emerging Enteropathogen Closely Related to *Escherichia coli*

**DOI:** 10.1093/gbe/evv211

**Published:** 2015-11-03

**Authors:** Tadasuke Ooka, Yoshitoshi Ogura, Keisuke Katsura, Kazuko Seto, Hideki Kobayashi, Kimiko Kawano, Eisuke Tokuoka, Masato Furukawa, Seiya Harada, Shuji Yoshino, Junji Seto, Tetsuya Ikeda, Keiji Yamaguchi, Kazunori Murase, Yasuhiro Gotoh, Naoko Imuta, Junichiro Nishi, Tânia A. Gomes, Lothar Beutin, Tetsuya Hayashi

**Affiliations:** ^1^Department of Microbiology, Graduate School of Medical and Dental Sciences, Kagoshima University, Japan; ^2^Department of Bacteriology, Faculty of Medical Sciences, Kyushu University, Fukuoka, Japan; ^3^Division of Microbiology, Department of Infectious Diseases, Faculty of Medicine, University of Miyazaki, Japan; ^4^Division of Bacteriology, Osaka Prefectural Institute of Public Health, Osaka, Japan; ^5^Center for Animal Disease Control and Prevention, National Institute of Animal Health, Ibaraki, Japan; ^6^Department of Microbiology, Miyazaki Prefectural Institute for Public Health and Environment, Miyazaki, Japan; ^7^Division of Microbiology, Kumamoto Prefectural Institute of Public Health and Environmental Science, Kumamoto, Japan; ^8^Department of Microbiology, Yamagata Prefectural Institute of Public Health, Yamagata, Japan; ^9^Department of Infection Diseases Bacteriology, Hokkaido Institute of Public Health, Hokkaido, Japan; ^10^Departamento de Microbiologia, Imunologia e Parasitologia, Universidade Federal de São Paulo—Escola Paulista de Medicina, Brazil; ^11^National Reference Laboratory for *Escherichia coli*, Federal Institute for Risk Assessment (BfR), Berlin, Germany

**Keywords:** *Escherichia albertii*, emerging enteropathogen, core genome, genomic comparison, interspecies horizontal gene transfer, detection system

## Abstract

*Escherichia albertii* is a recently recognized close relative of *Escherichia coli*. This emerging enteropathogen possesses a type III secretion system (T3SS) encoded by the locus of enterocyte effacement, similar to enteropathogenic and enterohemorrhagic *E. coli* (EPEC and EHEC). Shiga toxin-producing strains have also been identified. The genomic features of *E. albertii*, particularly differences from other *Escherichia* species, have not yet been well clarified. Here, we sequenced the genome of 29 *E. albertii* strains (3 complete and 26 draft sequences) isolated from multiple sources and performed intraspecies and intragenus genomic comparisons. The sizes of the *E. albertii* genomes range from 4.5 to 5.1 Mb, smaller than those of *E. coli* strains. Intraspecies genomic comparisons identified five phylogroups of *E. albertii*. Intragenus genomic comparison revealed that the possible core genome of *E. albertii* comprises 3,250 genes, whereas that of the genus *Escherichia* comprises 1,345 genes. Our analysis further revealed several unique or notable genetic features of *E. albertii*, including those responsible for known biochemical features and virulence factors and a possibly active second T3SS known as ETT2 (*E. coli* T3SS 2) that is inactivated in *E. coli*. Although this organism has been observed to be nonmotile in vitro, genes for flagellar biosynthesis are fully conserved; chemotaxis-related genes have been selectively deleted. Based on these results, we have developed a nested polymerase chain reaction system to directly detect *E. albertii*. Our data define the genomic features of *E. albertii* and provide a valuable basis for future studies of this important emerging enteropathogen.

## Introduction

The genus *Escherichia* belongs to the family *Enterobacteriaceae* and consists of three species (*Escherichia coli*, *Escherichia fergusonii*, and *Escherichia albertii*) and five cryptic clades (*Escherichia* C-I to C-V [Walk et al. 2009]). Among these species and clades, *E. coli* has been most intensively studied in a wide range of research fields, such as genetics, biochemistry, molecular biology, and biotechnology. In medical microbiology, the pathogenic *E. coli* is frequently associated with a range of intestinal and extraintestinal diseases in humans and animals. *Escherichia fergusonii* is implicated as an opportunistic extraintestinal pathogen of humans, birds, and mammals; however, clear evidence for the enteropathogenic nature of this species has not been found (Gordon 2013). *Escherichia* clades C-I to C-V are primarily recovered from environmental sources and their pathogenic potentials are unknown (Walk et al. 2009; Luo et al. 2011). In contrast, *E. albertii* has recently been recognized as a human enteropathogen and an avian pathogen responsible for epidemic mortality (Huys et al. 2003; Oaks et al. 2010). A substantial proportion of strains that have been identified as enteropathogenic *E. coli* (EPEC) were recently shown to be *E. albertii* (Ooka et al. 2012). This pathogen also causes outbreaks of gastroenteritis (Ooka et al. 2013) and may produce Shiga toxin (Stx2a and Stx2f) (Ooka et al. 2012; Murakami et al. 2014; Brandal et al. 2015). While the complete genome sequence of *E. albertii* strain KF1 was very recently reported (Fiedoruk et al. 2014), the genomic features, repertoire of virulence factors, and virulence mechanisms of *E. albertii* have not yet been characterized. Moreover, no large-scale genomic comparison of multiple *E. albertii* strains has been carried out and genomic differences between *E. albertii* and other *Escherichia* species (and clades) have not yet been well elucidated.

Here, we determined the complete genome sequences of three *E. albertii* strains and the draft sequences of additional 26 strains; we also performed robust intraspecies and intragenus genomic comparisons. Our analysis identified the core genome of *E. albertii*, the accessory genome specific to *E. albertii*, and a possible core genome of the genus *Escherichia*. Several unique or notable genetic features of *E. albertii* have also been identified. These data provide insights into the genomic evolution, adaptation, and virulence mechanisms of *E. albertii*. A nested polymerase chain reaction (PCR)-based *E. albertii*-specific detection system has also been developed. This detection system could be widely used to screen feces, food, and environmental samples for *E. albertii* strains.

## Materials and Methods

### Bacterial Strains, Culture Conditions, and DNA Purification

The strains used in this study are listed in supplementary table S1, Supplementary Material online. Bacteria were routinely grown in Lysogeny broth (LB, Difco) at 37°C with shaking. Genomic DNA was purified from 2 ml overnight cultures of each strain using a DNeasy Blood and Tissue kit (Qiagen) following the manufacturer’s instructions.

### Determination of the Complete Genome Sequences of Three *E. albertii* Strains and Gene Prediction and Annotation

The genomes of the three *E. albertii* strains CB9786, NIAH_Bird_3, and EC06-170 were sequenced using the Roche 454 GS FLX Titanium platform, 400–500 bp shotgun fragments and 8 kb-span paired end libraries. The sequence reads were assembled with GS Assembler ver. 2.3, and gaps were filled by sequencing fosmid clones and PCR products that spanned the gaps using a capillary sequencer (ABI3730). The three strains were resequenced with the Illumina MiSeq platform to correct sequencing errors made by the Roche 454. The protein-coding sequences (CDSs) and functional annotations were predicted using the Microbial Genome Annotation Pipeline (MiGAP; http://www.migap.org, last accessed November 12, 2015). Manual curation was performed using the in silico Molecular Cloning Genomics Edition software (IMC-GE; In Silico Biology, Inc.).

### Draft Genome Sequencing of 26 *E. albertii* Strains

The draft genome sequences of 26 *E. albertii* strains were generated using the Illumina MiSeq platform and 250–300 bp shotgun fragment libraries for each strain, which were prepared using the Nextera XT DNA Sample Prep kit (Illumina) following the manufacturer’s instructions. Sequencing reads were assembled using Platanus version 1.1.4 (Nikaido et al. 2013). The sequencing and assembling statuses of each strain are summarized in supplementary table S1, Supplementary Material online.

### Genome-Wide Phylogenetic Analyses

In addition to the 29 *E. albertii* strains (3 complete and 26 draft genomes) sequenced in this study, 5 *E. albertii* genomes (one complete and four draft sequences), 44 *E. coli* strains with completely sequenced genomes, 5 *E. fergusonii* strains (one complete and four draft genome sequences), and 15 draft genome sequences of *Escherichia* species belonged to cryptic clades were used (listed in supplementary table S2, Supplementary Material online; these complete and draft sequences of *E. fergusonii* and cryptic clades were obtained from the databases of NCBI or Broad Institute). Phylogenetic trees were created based on the concatenated sequences of 111 single copy genes that are fully conserved in all of the 98 strains. To select the 111 genes, we first performed a tBLASTn search in each of above-mentioned genomes using all CDSs of the *E. albertii* strain CB9786 as queries (at cutoff values of 80% amino acid sequence identity and 100% length matches). Based on this analysis, we identified single-copy genes that are perfectly conserved in all examined genomes. The pairwise homoplasy index (PHI) test (Bruen et al. 2006) was then performed to select genes with a low probability of recombination (at a cutoff value of *P* < 0.05). Finally, a neighbor-joining tree was constructed using the SplitsTree 4 software (Huson and Bryant 2006).

### Gene Repertoire Comparisons between *E. albertii, E. coli*, and *E. fergusonii*

All CDSs of the three *E. albertii* strains or the 44 fully sequenced *E. coli* strains were classified into 4,931 or 19,274 CDS clusters, respectively, using the CD-HIT algorithm (at cutoff values of 90% sequence identity and 60% aligned length coverage; Fu et al. 2012). The genes (or gene families) shared by the *E. coli* and *E. albertii* lineages and those specific to *E. albertii* were identified with a BLASTp analysis of the 4,931 *E. albertii* CDS clusters against the 19,274 *E. coli* CDS clusters using an 80% cutoff for sequence identity and a 60% cutoff for aligned length coverage. The results were converted into binary scores (present = 1 or absent = 0). Hierarchical clustering of the 34 *E. albertii* strains based on their gene repertoires was performed using the Cluster 3.0 software (Eisen et al. 1998).

### Analyses of LEE Elements and LEE-Dependent T3SS Effectors in *E. albertii* Genomes

Contigs or scaffolds that contained locus of enterocyte effacement (LEE) core regions were extracted from each of the 30 *E. albertii* draft genome sequences and genes in the LEE core region were manually annotated using the IMC-GE software. To investigate the LEE-dependent type III secretion system (T3SS) effector repertoires of *E. albertii*, the 31 genome sequences of *E. albertii* strains (other than the three *E. albertii* strains fully sequenced and annotated in this study) were analyzed with BLASTx using the T3SS effectors that have been identified in the enterohemorrhagic *E. coli* (EHEC) O157 strain Sakai (Hayashi et al. 2001), the EPEC O127:H6 strain E2348/69 (Iguchi et al. 2009), and the EPEC O111:H- strain B171 (Ogura et al. 2008) as queries, with filtering by hit length coverage (>70%) and sequence identity (>30%).

### RT-PCR Analysis

Bacterial cells were harvested from 2 ml cultures at the logarithmic phase (OD_600_≈0.8). The cultures were grown in Dulbecco’s Modified Eagle Medium (DMEM) medium (Gibco) or tryptone water containing 1.5% Bacto Tryptone (Difco) and 0.5% sodium chloride at 37°C. Total RNA was extracted using the RNAprotect Bacteria Reagent and RNeasy Plus Mini Kit (Qiagen) according to the manufacturer’s instructions. The RNA samples were treated with DNase and purified using the RNeasy MiniElute Cleanup kit (Qiagen). RT-PCR reactions were carried out using 1 µg of purified RNA and the SuperScript II One Step RT-PCR system with Platinum Taq High Fidelity (Invitrogen) following the manufacturer’s instructions. The primer sets and PCR conditions employed are described in the legend of supplementary figure S3*F*, Supplementary Material online.

### Motility Assay

Logarithmic-phase bacterial cultures (OD_600_≈0.8), which were grown in LB, DMEM medium, or tryptone water at two different temperatures (37°C and 42°C), were stabbed into tryptone agar plates (0.3% agarose with tryptone water) using toothpicks. After incubating for 20 h at the same temperature used for precultivation, the motilities of each strain were examined.

### Ultrastructural Studies

For ultrastructural studies used to detect flagella structures, bacterial cells were grown under the same conditions as those used for the motility assays. The cells were then fixed using 4% paraformaldehyde for 8 h and washed with 0.1 M phosphate buffer (pH 7.4), followed by negative staining with 1% phosphotungstic acid (pH 7.4) on carbon-Formvar copper grids. The samples were examined using a Hitachi S4800 transmission electron microscopy at 80 kV.

### Development of a Nested PCR System to Specifically Detect *E. albertii*

To identify the target sequences for the specific detection of *E. albertii*, we first identified sequences in the strain CB9786 that are not conserved in any of the 60 complete or draft genome sequences of other *Escherichia* species/clades that are listed in supplementary table S2, Supplementary Material online, by a BLASTn search (with an 80% cutoff for nucleotide sequence identity and a 100-bp cutoff for alignment length). After excluding these sequences, the remaining CB9786 genome sequences were subjected to a BLASTn analysis to identify sequences that are conserved in all *E. albertii* strains used in this study (>90% nucleotide identity with >100 bp alignment length). Among the *E. albertii*-specific sequences identified in this analysis, we selected a region encompassing EACBF2103 and EACBF2104 (CDS numbers in strain CB9786) and designed two primer pairs for a nested PCR system (PCR primers and PCR conditions are described in the legend of supplementary fig. S4, Supplementary Material online).

## Results and Discussion

### General Genomic Features of *E. albertii*

We determined the complete genome sequences of three *E. albertii* strains (NIAH_Bird_3, EC06-170, and CB9786) that were among the 27 strains that we previously identified (Ooka et al. 2012). NIAH_Bird_3 was isolated from the feces of a bird (*Puffinus tenuirostris*); the other two strains were clinical isolates. CB9786 was isolated in Germany. The other two strains were isolated in Japan. The draft genome sequences of 26 *E. albertii* strains were also determined; 22 strains, which included two *stx2f*-positive strains, were also among the previously identified 27 strains (Ooka et al. 2012). One strain was isolated in an outbreak in Japan (Ooka et al. 2013). Three strains were newly identified clinical isolates. Additional strain information and the sequencing status of each strain are available in supplementary table S1, Supplementary Material online.

The general genomic features of three fully sequenced strains are summarized in table 1. The chromosomes were approximately 4,600 kb in size, and the GC contents were all 49.8%. None of the strains possessed any plasmids. The estimated genome sizes of the 26 *E. albertii* draft sequences, which may include plasmid sequences in some strains, ranged from 4,511 to 5,121 kb; the median genome size was 4,777 kb (supplementary table S1, Supplementary Material online). Because the sizes of the 44 fully sequenced *E. coli* chromosomes (supplementary table S2, Supplementary Material online) range from 4.6 to 5.6 Mb (median 5,132 kb), the chromosomes of *E. albertii* appear to be smaller than those of *E. coli* and similar in size to those of *E. fergusonii* (median 4,711 kb).

**Table 1:** General Genomic Features of the Three Fully Sequenced *Escherichia albertii* Strains

Strain	CB9786	NIAH_Bird_3	EC06-170
Chromosome (bp)	4,598,983	4,560,575	4,657,167
CDSs^a^	4,284 (45)	4,125 (43)	4,275 (41)
rRNA operons	7	7	7
tRNA genes^b^	89	86	97
PPs	7	4	5
IEs^c^	8	7	7
IS elements	8	7	11

^a^Number of pseudogenes are shown in parentheses.

^b^Among these, 75 are shared by three *E. albertii* strains.

^c^Including the LEE element.

**Fig. 1.—:**
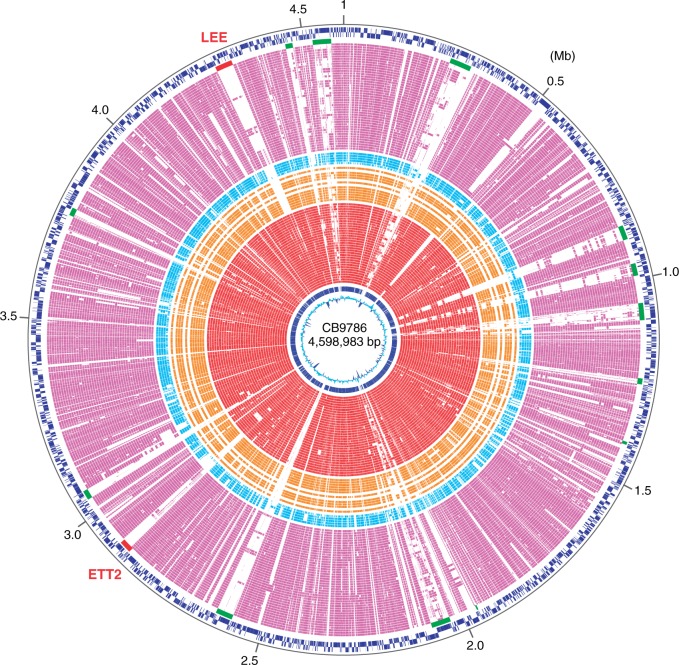
Circular presentation of the genome of *Escherichia albertii* strain CB9786 and conservation of CB9786 genes in the genomes of *E. albertii*, *E. fergusonii*, *E. coli*, and other *Escherichia* species. From the outside in: nucleotide positions (in Mb); CDSs transcribed clockwise and counterclockwise, respectively; locations of PPs and IEs (green) and LEE and ETT2 (red); CDSs (purple) conserved in the *E. coli* strains NA114, SE15, S88, APEC O1, IHE3034, UTI89, UM146, ED1a, CFT073, ABU_83972, LF82, NRG857C, 536, E2348/69, SMS-3-5, CE10, IAI39, UMN026, 042, E24377A, KO11FL, 55989, 2011C-3493, 2009EL-2071, 2009EL-2050, SE11, IAI1, O103_12009, O111_11128, O26_11368, APECO78, HS, ATCC8739, P12b, MG1655, H10407, UMNK88, O157_Xuzhou21, O157_EC4115, O157_EDL933, O157_TW14359, O157_Sakai, O55_RM12579, and O55_CB9615; CDSs (light blue) conserved in the *E. fergusonii* strains GTA-294-5-RBA-P2, ECD227, B253, GTA-1753-4-RBA-P5, and ATCC35469; CDSs (orange) conserved in the *Escherichia* cryptic clade strains TW10509 (C-I), TW09276, KTE31, KTE114 (C-III), H605 (C-IV), KTE96, KTE11, E1118, HT073016, KTE52, and KTE159 (C-V); CDSs (red) conserved in the *E. albertii* strains HIPH08472, EC03-127, 24, 20H38, NIAH_Bird_23, K7394, EC05-44, TW08933, NBRC 107761, TW07627, KF1, CB10113, B156, 4051-6, EC03-195, EC05-81, K7744, NIAH_Bird_16, E2675, NIAH_Bird_8, NIAH_Bird_2, NIAH_Bird_24, NIAH_Bird_13, 94389, EC05-160, KU20110014, EC06-170, NIAH_Bird_3, CB9791, NIAH_Bird_26, NIAH_Bird_5, NIAH_Bird_25, and K7756; CDSs (blue) conserved in the three *E. albertii* strains fully sequenced in this study; and G+C content.

Seven rRNA operons were identified in the three fully sequenced *E. albertii* strains; the same number of rRNA operons were found in *E. coli* and *E. fergusonii*. The number of tRNA genes ranged from 86 to 97. Some of these genes were located in prophage (PP) regions, as seen in *E. coli* (possessing 79-90 tRNA genes) and *E. fergusonii* (87 genes in strain ATCC35469). Various sizes of strain-specific insertions were present throughout the chromosome. Most of these insertions were PPs, integrative elements (IEs), and insertion sequence (IS) elements (fig. 1 and supplementary tables S3 and S4, Supplementary Material online). The three *E. albertii* strains contain a similar set of PPs and IEs, but remarkable structural and sequence diversities were observed in each PP/IE family, particularly in PP families (supplementary fig. S1*A*, Supplementary Material online). Notably, the three *E. albertii* strains (7–11 copies) contained significantly fewer IS elements than in *E. coli* (42–224 copies) or *E. fergusonii* (29 copies) (Touchon et al. 2009). Because IS elements play important roles in the diversification of bacterial genomes via various mechanisms, including transpositional gene inactivation (Ooka et al. 2009; Darmon and Leach 2014), the relatively small number of pseudogenes (41–45 genes) in the three *E. albertii* strains may be partly attributable to the small number of IS elements.

### Phylogenic Relationship, Genomic Synteny, and Nucleotide Sequence Identity between *E. albertii, E. coli*, and *E. fergusonii*

**Fig. 2.—:**
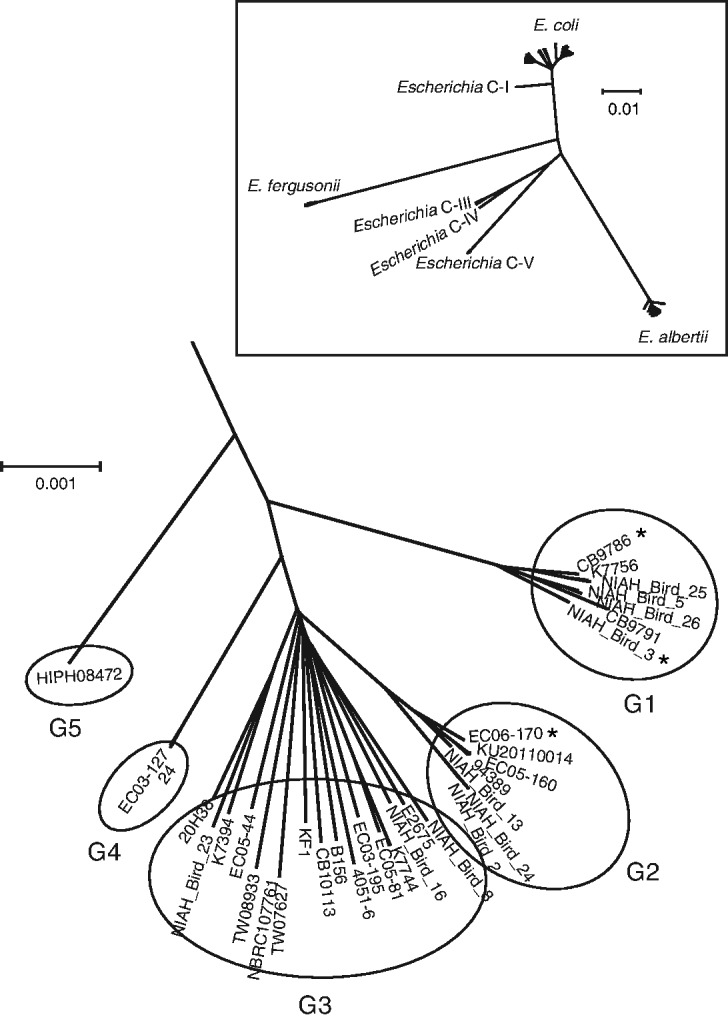
Genome-wide phylogenetic analysis of *Escherichia albertii* strains and those belonging to other *Escherichia* species and clades. A neighbour-joining tree (in box) was constructed using the concatenated nucleotide sequences of 111 single copy genes that are fully conserved in the genomes of 34 *E. albertii* strains, 44 *E. coli* strains, 5 *E. fergusonii* strains, and 15 strains belonging to *Escherichia* cryptic clades with a low probability of recombination. An enlarged view of the *E. albertii* lineage is shown. The three strains fully sequenced in this study are indicated by asterisks. G1–G5 indicate five phylogroups of *E. albertii*.

To investigate the precise phylogenetic relationship of *E. albertii* with other *Escherichia* species and clades, we selected 111 single copy genes that are highly conserved in all 34 *E. albertii* strains, the 44 fully sequenced *E. coli* strains, 5 *E. fergusonii* strains (one complete and four draft), and 15 draft genomes of other *Escherichia* clades (>80% amino acid sequence identity and 100% length match with a low probability of recombination). A neighbor-joining tree constructed using the concatenated sequences of these genes indicated that *E. albertii* strains form a lineage distinct from other *Escherichia* species and clades (fig. 2 and supplementary fig. S1*B*, Supplementary Material online) as recently reported by Luo et al. (2011). *Escherichia albertii* strains were further divided into five phylogroups (G1–G5), with G3 containing more divergent members than the other phylogroups (fig. 2).

The overall gene organization on chromosome was well conserved between the three fully sequenced *E. albertii* strains (supplementary fig. S1*C*, Supplementary Material online). The average nucleotide identity (ANI) was greater than 98% among these strains (ranging from 98.2% to 99.2%). While overall colinearity was observed between *E. albertii* and *E. coli* chromosomes (K-12 MG1655 was used to represent *E. coli* in supplementary fig. S1*C*, Supplementary Material online), the ANI values between the three *E. albertii* strains and the ten fully sequenced *E. coli* strains that we selected from each of the five *E. coli* phylogroups (A, B1, B2, D, and E; two strains from one phylogroup as listed in supplementary table S2, Supplementary Material online) were 89.2–90.1%. Five *E. fergusonii* strains exhibited 86.4–86.9% and 88.8–89.3% ANIs to *E. albertii* and *E. coli* strains, respectively; remarkable genomic rearrangement was also observed between *E. fergusonii* ATCC35469 and three fully sequenced *E. albertii* strains (supplementary fig. S1*C*, Supplementary Material online). Additionally, ANI values between the three *E. albertii* and the strains of *Escherichia* cryptic clades (C-I, C-III, C-IV, and C-V) were 89.2–89.4%, 89.4–89.7%, 89.5–89.7%, and 89.0–89.5%, respectively. The low ANI values observed between *E. albertii* and all other *Escherichia* species and clades support a notion that *E. albertii* is phylogenetically distinct from other *Escherichia* species and clades (Walk et al. 2009; Luo et al. 2011).

### Intraspecies Gene Repertoire Comparison among *E. albertii* Strains

**Fig. 3.—:**
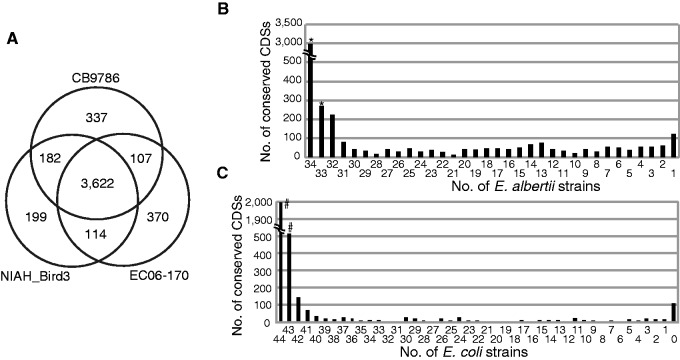
Intraspecies and interspecies conservation of *Escherichia albertii* genes. (*A*) A Venn diagram showing the number of unique or shared CDSs among the three completely sequenced *E. albertii* strains. (*B*) Distribution of 3,622 CDSs that are shared by three fully sequenced *E. albertii* strains among the 34 strains analyzed in this study. The CDSs indicated by asterisks are conserved in all or 33 strains. (*C*) The presence of homologs for 3,622 CDSs shared by three fully sequenced *E. albertii* strains among 44 fully sequenced *E. coli* strains. The CDSs indicated by asterisks are conserved in all or 43 of the *E. coli* strains.

To analyze the gene repertoires of *E. albertii* strains, we first clustered all CDSs identified in the three complete *E. albertii* genomes (12,684 in total) using the CD-HIT algorithm (cutoffs at 90% amino acid sequence identity and 60% aligned length coverage) and obtained 4,931 CDS clusters. Among these clusters, 3,622 were shared among the three strains (fig. 3*A*). A tBLASTn analysis of the shared CDSs in the genome sequences of 34 *E. albertii* strains identified 3,250 CDS clusters that are conserved in all (2,981 clusters) or 33 (269 clusters) of the 34 *E. albertii* strains; these clusters likely represent the core CDSs of *E. albertii* (fig. 3*B*). Hierarchical clustering of the 34 strains based on their gene repertoires indicated that the gene repertoire encoded on nonmobile genetic element regions correlates with the phylogeny of each strain. A few strains that belong to phylogroup G3, which contains diverse strains, were exceptions to this rule. When CDSs on PPs and IEs were included in the analysis, several strains belonging to other phylogroups did not follow the phylogeny (supplementary fig. S1*D* and *E*, Supplementary Material online). This finding suggests that the horizontal gene transfer mediated by these mobile genetic elements has played significant roles in the diversification of the gene repertoire of *E. albertii* strains beyond phylogeny.

### Interspecies Gene Repertoire Comparison between *E. albertii*, *E. coli*, and *E. fergusonii*

The clustering analysis of all CDSs encoded in the 44 fully sequenced *E. coli* genomes using CD-HIT, with the same cutoff values as used for *E. albertii*, generated 19,274 CDS clusters. A comparison of the gene repertories between the 34 *E. albertii* and 44 *E. coli* strains revealed that 2,511 out of the 3,250 core *E. albertii* CDS clusters are highly conserved in *E. coli* (present in all or 43 strains) (fig. 3*C*). By comparing the core *E. albertii* CDSs with the 5 *E. fergusonii* strains, 1 *Escherichia* C-I strain, 3 *Escherichia* C-III strains, 1 *Escherichia* C-IV strain, and 6 *Escherichia* C-V strains, we identified 1,601, 2,989, 2,941, 2,971, and 2,868 CDS clusters that are conserved in the strains belonged to each species/clade, respectively. Among these clusters, 1,345 were highly conserved in all *Escherichia* strains analyzed, and thus likely represent the core CDSs of the genus *Escherichia*.

Based on this analysis, we identified 55 *E. albertii* species-specific CDSs that are highly conserved in *E. albertii* but absent or divergent in sequence in all other *Escherichia* species and clades; therefore, such sequences confer specific features to *E. albertii* (supplementary table S5*A*, Supplementary Material online). Among these CDSs, some functions have been assigned to 29 CDSs, such as genes related to energy production (fumarate reductase subunits, electron transfer flavoproteins, etc.) and metabolism (alpha-amylase, phosphotransferase system components, etc.), as well as genes for several transporters and potential virulence factors (a cytolethal distending toxin belonging to the II/III/IV subtype and several T3SS-related proteins).

We also identified 95 CDSs that were conserved in 44 *E. coli* strains but not in any *E. albertii* strains (supplementary table S6, Supplementary Material online). By comparing gene repertoires among the three completely sequenced *E. albertii* strains and the *E. coli* strain K-12 MG1655, we identified a large number of operons or gene clusters that are absent in the *E. albertii* lineage (supplementary table S6, Supplementary Material online). They included operons/gene clusters related to known biochemical properties that help to discriminate *E. albertii* from *E. coli*, such as the negative-fermentation of lactose, xylose, and raffinose and the inability to produce β-glucuronidase (Ooka et al. 2012; Nimri 2013; Murakami et al. 2014). All 34 *E. albertii* strains analyzed in this study lack the genes responsible for these metabolic functions; the *lacA/Y/I* genes (related to the lactose utilization; the *lacZ* gene remains conserved, but its physiological function is unknown) and the *xylBAFGHR* (xylose utilization), *melRAB* (raffinose utilization), and *uidCBAR* (β-glucuronidase production) loci are absent in *E. albertii* (supplementary fig. S1*F*, Supplementary Material online). The *rha* operon for rhamnose formation is missing in all *E. albertii* strains. Because most *E. coli* strains ferment rhamnose, the inability to ferment rhamnose could serve as a biochemical marker for *E. albertii*. In our previous study (Ooka et al. 2012), the *E. albertii* strain NIAH_Bird_23 could weakly ferment lactose. This strain lacks the *lacA/Y/I* genes, like other *E. albertii* strains. This finding suggests that NIAH_Bird_23 may possess an unknown pathway for lactose fermentation. In addition, the *betIAB* operon for glycine betaine synthesis that is required for the stress response to high-osmolality environments (Lamark et al. 1996) is also missing in all *E. albertii* strains. Further analysis is required, but there may be two possibilities that *E. albertii* possesses an alternative osmotic protection and that *E. albertii* is more sensitive to osmotic stress than *E. coli*.

### LEE and LEE-Related T3SS Effectors

The LEE pathogenicity island (∼35 kb in length) encodes a T3SS machinery, chaperones, and several effectors (Wong et al. 2011). In addition to *E. coli* (EPEC and EHEC) and *Citrobacter rodentium*, *E. albertii* is known to possess LEE (Hyma et al. 2005). The core region of LEE is also highly conserved in *E. albertii*; it has been integrated into the tRNA-*pheU* gene in all *E. albertii* strains (data not shown).

EHEC and EPEC produce a large number of non-LEE effectors that are secreted by the LEE-encoded T3SS. Many of these effectors are encoded by PPs and IEs (Tobe et al. 2006; Deng et al. 2010). While *E. albertii* contained significantly fewer PPs and IEs than EHEC and EPEC, most *E. albertii* strains contain a high number of LEE-encoded T3SS-dependent effectors (38 genes in average [19–51 genes]), making *E. albertii* effector repertoires similar to those of EPEC and EHEC (supplementary fig. S2*A*, Supplementary Material online). The three fully sequenced *E. albertii* genomes contained a total of 15 non-LEE effector-encoding loci. Five of these loci were on PPs, and three were on IEs. However, seven other loci were in chromosomal regions not related to PPs or IEs. This distribution sharply contrasts those of EHEC and EPEC (Iguchi et al. 2009; Ogura et al. 2009) (supplementary fig. S2*B*, Supplementary Material online).

### Presence of a Complete *E. coli* T3SS 2

**Fig. 4.—:**
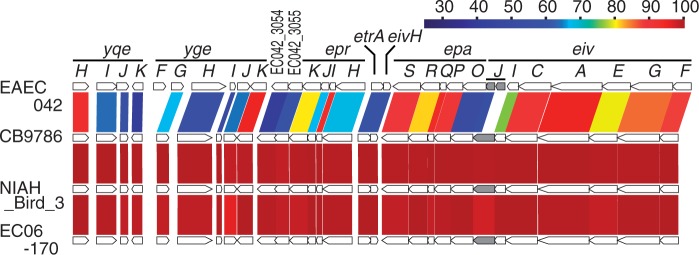
Structures of the *Escherichia albertii* ETT2 (*E. coli* T3SS 2) region. Gene organizations of the ETT2 regions of three fully sequenced *E. albertii* strains are shown. For comparison, the ETT2 region of the enteroaggregative *E. coli* strain 042 is also shown. Note that the ETT2 region has been highly degraded in most *E. coli* strains.

An *E. coli* T3SS 2 (ETT2)-like genomic island has been integrated into the tRNA-*glyU* gene in the genomes of the three completely sequenced *E. albertii* strains. ETT2 is a cryptic second T3SS in the *E. coli*/*Shigella* lineage (Hayashi et al. 2001; Ren et al. 2004; Ideses et al. 2005) that is distantly related to the *Salmonella* T3SS encoded on *Salmonella* pathogenicity island 1. In *E. coli*/*Shigella*, the ETT2 region was found at the tRNA-*glyU* locus; however, this region has been deeply degraded in most strains. Among *E. coli*/*Shigella* strains, only the EAEC strain 042 appears to possess a nearly complete ETT2 region. In this strain, one gene (*eivJ*) is disrupted by a frameshift mutation. In contrast, the ETT2 regions in all three of the completely sequenced *E. albertii* strains are apparently intact. In these strains, *eivJ* has not been disrupted (fig. 4). The presence of ETT2 in *E. albertii* indicates that ETT2 was acquired by the *E. albertii*/*E. coli* lineage before the separation of the two species. In our preliminary analysis using strains CB9786 and NIAH_Bird_3, ETT2 gene expression (*eivF* and *eprH*) was detected by RT-PCR (supplementary fig. S3*F*, Supplementary Material online). In addition, 13 of the other 31 *E. albertii* strains analyzed in this study also possess intact ETT2 regions (supplementary fig. S3*A*, Supplementary Material online). Intriguingly, six of the remaining 18 strains contain a single frameshift mutation in the *eivJ* gene. Therein, a 1-bp deletion or insertion has occurred in the same poly(A) sequence (supplementary fig. S3*B*, Supplementary Material online). Therefore, programmed ribosomal frameshifting or transcriptional realignment may produce an intact EivJ protein in these six strains (Sharma et al. 2014). The well-conserved structure of ETT2 in *E. albertii* suggests a possibility that the ETT2-encoded T3SS may be involved in the pathogenicity of this enteropathogen. However, further confirmation of gene expression and functional analyses are required.

### Genes for Flagellar Biosynthesis and Chemotaxis

**Fig. 5.—:**
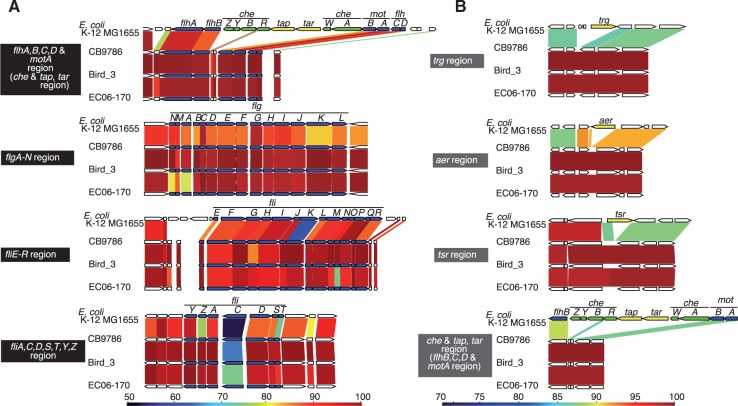
Gene organizations of genomic loci encoding flagellar biosynthesis- and chemotaxis-related genes. Gene organizations of genomic loci encoding flagellar biosynthesis- and chemotaxis-related genes are shown. For comparison, analogous loci from *Escherichia coli* K-12 MG1655 are shown. While amino acid sequence identities between orthologous genes are presented in panel *A*, the nucleotide sequence identities of conserved genomic regions are presented in panel *B* to illustrate the specific deletion of chemotaxis-related genes. The *flhA-flhD* locus is presented in both panels *A* and *B*.

*Escherichia albertii* is known to be nonmotile (Oaks et al. 2010; Ooka et al. 2012; Nimri 2013; Murakami et al. 2014). However, gene clusters known to be required for flagellar biosynthesis in *E. coli* are fully conserved in the three fully sequenced *E. albertii* strains (fig. 5*A*). The transcriptional regulators for flagellar biosynthesis are also conserved. In addition, our preliminary RT-PCR analysis of the genes indicated that *flhA* and *fliD* in the flagellar operons are transcribed in strains CB9786 and NIAH_Bird_3 (supplementary fig. S3*F*, Supplementary Material online). Curiously, the genes encoding chemotaxis-related proteins (CheA-Z), four methyl accepting chemotaxis proteins, and an aerotaxis receptor protein (Aer) are selectively missing (fig. 5*B*). An analysis of other 31 *E. albertii* strains revealed that at least 23 strains (74%) possess complete gene sets for flagellar biosynthesis and regulation; none of these 31 strains contain chemotaxis-related genes (supplementary fig. S3*E*, Supplementary Material online). Although the genes that encode most proteins related to flagellar biosynthesis and its regulation are highly conserved in sequence (>90% identity) among the 34 strains, the *fliC* gene, which encodes flagellin, shows remarkable sequence diversity (65–85%) (supplementary fig. S3*C* and *D*, Supplementary Material online). Therefore, the *fliC* gene appears to be under immunological selection in hosts. Although we examined the cell surface of *E. albertii* strains grown in various conditions by electron microscopy, flagella-like structures were not detected. However, our data suggest that flagella-related genes in *E. albertii* are active and *E. albertii* may express some flagella-related surface structures in hosts.

### Identification of Species-Specific Sequences and Development of a Nested PCR-Based *E. albertii* Detection System

A selective medium for *E. albertii* is currently not available. Three PCR systems to identify *E. albertii* and differentiate it from *E. coli* have been developed thus far (Hyma et al. 2005; Maeda et al. 2014; Smati et al. 2015). However, these systems detect sequence variations in housekeeping genes between *E. albertii* and *E. coli*. We require more specific and sensitive PCR systems that can directly detect and systematically screen for *E. albertii* in various samples, such as food, water, and human and animal feces. We attempted to develop such a PCR system based on the obtained genome sequence information.

To this end, we identified 118 *E. albertii* species-specific sequences (71,280 bp in total; ranging from 100 to 4,068 bp) (supplementary table S5*B*, Supplementary Material online) by comparing the genome sequences of 34 *E. albertii* strains, 44 fully sequenced *E. coli* strains, 5 *E. fergusonii* strains and 11 strains from *Escherichia* clades C-I to C-V. Among these sequences, we selected one target sequence inserted between the *yejH* and *yejK* genes. This sequence encodes *E. albertii*-specific fumarate reductase subunits and a C-type cytochrome. Using this information, we designed two pairs of primers for a nested PCR system (supplementary fig. S4*A*, Supplementary Material online). We have confirmed that both PCR primer pairs for the first and second PCR yielded expected PCR products from all *E. albertii* strains used in this study; none of the *E. coli* strains yielded PCR products (supplementary fig. S4*B*, Supplementary Material online). This system could be used for a wide range of investigations. We have successfully adopted this nested PCR system to screen the feces of various animals for *E. albertii* (data not shown).

## Supplementary Material

Supplementary figures S1–S4 and tables S1–S6 are available at *Genome Biology and Evolution* online (http://www.gbe.oxfordjournals.org/).

Supplementary Data
