# Supplementary material for: Defining the Genome Features of Escherichia albertii, an Emerging Enteropathogen Closely Related to Escherichia coli
Source: Genome Biol Evol. 2015 Nov 3;7(12):3170–9. doi: 10.1093/gbe/evv211 (PMC4700944; doi:10.1093/gbe/evv211)
Supplement: Supplementary Data [file supp_7_12_3170__index.html]

Defining the Genome Features of Escherichia albertii, an Emerging Enteropathogen Closely Related to Escherichia coli — Supplementary Data 

# Defining the Genome Features of *Escherichia albertii*, an Emerging Enteropathogen Closely Related to *Escherichia coli*

## Supplementary Data

files

- Supplementary Data - docx file
- Supplementary Data - pdf file
- Supplementary Data - pdf file
