# Supplementary material for: Defining the Genome Features of Escherichia albertii, an Emerging Enteropathogen Closely Related to Escherichia coli
Source: Genome Biol Evol. 2015 Nov 3;7(12):3170–9. doi: 10.1093/gbe/evv211 (PMC4700944; doi:10.1093/gbe/evv211)
Supplement: Supplementary Data [file supp_evv211_FIGURE_LEGENDS_FOR_SUPPLEMENTAL_FIGURES_GBEre.docx]

**FIGURE LEGENDS FOR SUPPLEMENTAL FIGURES**

**Fig. S1. Intra- and inter-species comparisons between *E. albertii* and *E. coli* and genome-wide phylogenetic analysis of strains of the genus *Escherichia***

(A) Dot-plot matrices of nucleotide sequence conservation between the PPs and IEs in the three fully sequenced *E. albertii* strains. (B) A neighbor-joining tree constructed using the concatenated nucleotide sequences of 111 single copy genes that are fully conserved in the genomes of 34 *E. albertii* strains, 44 *E. coli* strains, 5 *E. fergusonii* strains, and 15 strains from other *Escherichia* species with a low probability of recombination. (C) Dot-plot matrices of nucleotide sequence conservation between the chromosomes of three *E. albertii* strains completely sequenced in this study, the *E. coli* strain K-12 MG1655, and the *E. fergusonii* strain ATCC35469.  (D) Hierarchical clustering trees showing the gene repertoire similarity between *E. albertii* strains. Hierarchical cluster analysis was performed using the Cluster 3.0 software, and the result was visualized using the Java TreeView software. The lower tree ( “whole” ) was constructed using the all identified genes. Genes on mobile genetic elements (prophages and integrative elements) were excluded from the analysis shown in the upper panel ( “w/o PP&IE” ).  (E) Conservation of CDSs identified in each of the three *E. albertii* strains completely sequenced in this study with other sequenced *E. albertii* strains.  (F) Gene organizations of the *E coli* K-12 genomic loci responsible for xylose, raffinose, rhamnose, and lactose fermentation and β-glucuronidase production and analogous regions in the *E. albertii* genomes fully sequenced in this study.

**Fig. S2. LEE-related genomic information in *E. albertii***

(A) Repertoires of the LEE-encoded T3SS-dependent effectors of *E. albertii*, EHEC and EPEC strains (≥ 30% amino acid sequence identity and ≥ 60% length coverage). Gray boxes represent the presence of the genes. When two or more copies were present, the numbers of copies are indicated in the boxes. The numbers of pseudogenes are shown in parentheses. The three *E. albertii* strains completely sequenced in this study are indicated by asterisks. (B) Genomic locations of the genes for non-LEE encoded T3SS-secreted effector proteins in the three *E. albertii* strains completely sequenced in this study.

**Fig. S3. *E. coli* type III secretion system 2 (ETT2) and flagella-related genes in *E. albertii*.**

(A) Conservation of the ETT2 genes in 34 *E. albertii* strains. Black and gray boxes represent present and absent (or disrupted) genes, respectively. The *eivJ* genes that have been frame-shifted by 1-base deletions or insertions are indicated by blue or green boxes, respectively. The names of strains that contain an apparently intact ETT2 are in red; the names of strains containing 1-base deletions or insertions in *eivJ* are in blue or green, respectively.  (B) Nucleotide sequences of intact or frame-shifted *eivJ* genes from *E. albertii* strains. Only sequences encompassing a poly(A) sequence in which 1-base deletions or insertions were observed are shown.  (C) Sequence diversities of flagella-related genes among 34 *E. albertii* strains. Amino acid sequence identities of each gene among the 34 strains were calculated by pair-wise comparisons and the average sequence identities for each gene are presented. Vertical bars represent standard deviations.  (D) Phylogenetic analysis of *fliC* genes among *E. albertii* (red) and *E. coli* (black) strains. Forty-two *E. coli fliC* sequences available in public databases are included in this analysis; NJ and UPGMA trees are shown. Because the *E. coli* H52 allele clustered with *E. albertii fliC* genes in the NJ tree but not in the UPGMA tree, interspecies horizontal transfer of the *fliC* gene was not supported.  (E) Conservation of flagella- and chemotaxis-related genes in 34 *E. albertii* strains. Black and gray boxes represent present and absent (or disrupted) genes, respectively. Genes indicated by red boxes were not obtained from draft sequences.  (F) RT-PCR detection of the expression of *flhA* and *fliD* genes (flagellar biosynthesis) and *eivF* and *eprH* genes (ETT2) in the *E. albertii* strains CB9786 and NIAH_Bird_3. Each strain was grown in tryptone water or DMEM at 37 ˚C. The expression levels of *flhA* and *fliD* were detected in the two *E. albertii* strains under both culture conditions. The expression of *eivF* was detected in both strains cultured in tryptone water; expression was also detected in strain NIAH_Bird_3 cultured in DMEM. The expression of *eprH* genes was detected in strain CB9786 grown in tryptone water and strain NIAH_Bird_3 grown in DMEM. The *gyrB* gene was used as a constitutively expressed control gene. RNA samples from the *E. coli* strain K-12 MG1655 and O157 Sakai and genomic DNA from the *E. albertii* strain 4051-6 were analyzed as positive or negative controls. Note that the *E. albertii* strain 4051-6 contains the *fliD* gene and lacks the *flhA* gene. The following primers were used: flhA_RT_F (forward; 5’ -CGACGGCGGTTAATTCATGT-3’) and flhA_RT_RX (5’ - GCGAGCAGATGGTGAATCA-3’ ) for the detection of the *flhA* gene (product size: 900 bp); fliD_RT_F (5’ -GTGCTGTTTAACGAGGTCAT-3’ ) and fliD_RT_RX (5’ -CAGCAACACCATCAGCGAC-3’ ) for the detection of the *fliD* gene (product size: 648 bp); eivF_RT_F (5’ - GACTCTCCATCCACAGAGT-3’ ) and eivF_RT_RX (5’ - CAGAAGTTAACTCTGCTGGA-3’ ) for the detection of the *eivF* gene (product size: 552 bp); eprH_RT_F (5’ -GCATAAATACGTGCAGTCTCA-3’ ) and eprH_RT_RX (5’ -TGGAGACAGTACAGGCAGT-3’ ) for the detection of the *eprH* gene (product size: 522 bp); and gyrB_RT_F (5’ -CTGGAAGCCATCGTTCCAC-3’ ) and gyrB_RT_RX (5’ -TGGTAGATAACGCTATCGAC-3’ ) for the detection of the *gyrB* gene (product size: 662 bp). PCR amplification was achieved in a 50 μl reaction mix using SuperScript III RT/Platinum Taq enzyme (Invitrogen) according to the manufacturer’ s instructions. One microgram of RNA was used as the RNA template. PCR reactions were carried out using the following conditions: 37 ˚C for 30 min, 40 cycles of 2 min denaturation at 94 ˚C, 30 sec annealing at 54 ˚C, and 1 min elongation at 68 ˚C, and a final extension step of 5 min at 68 ˚C. One microliter of the reaction mixture of each sample was applied to agarose gels.

**Fig. S4. A nested PCR system to specifically detect *E. albertii*.**

(A) Locations of the 1st and 2nd primer pairs.  (B) Evaluation of the specificity of each primer pair using various *E. albertii* and *E. coli* strains. The primers for the 1st PCR are E_al_OF (5’ -GGTCCATAATGAATCTGACTGA-3’) and E_al_OR (5’ - CCATATGACAGGCGTAATTGAT-3’). The primers for the 2nd PCR are E_al_NF (5’ - CAGTCGATGGTTTCACCTGA-3’) and E_al_NR (5’ - ACACCGTGGCGAAATGGCA-3’). The product sizes for the 1st and 2nd PCR reactions were 846 bp and 731 bp, respectively. PCR amplification was carried out in 15-μl reactions using KAPA-taq extra (KAPABIOSYSTEMS) according to the manufacturer’ s instructions. One microliter of genomic DNA was used as the DNA template. PCR was carried out using the following conditions: 96 ˚C for 30 min, 30 cycles of 30 sec denaturation at 96 ˚C, 30 sec annealing at 54 ˚C (60 ˚C for the 2nd PCR), and 1 min elongation at 72 ˚C, and a final extension step of 5 min at 72 ˚C. Genomic DNA was prepared from a colony using the alkaline-boiling method as previously described (Ooka et al. 2009. J Clin Microbiol. 47(9):2888-2894). One microliter of the reaction mixture of each sample was applied to agarose gels. In addition to the 29 *E. albertii* strains sequenced in this study, the following 14 *E. coli* strains were selected from the five *E. coli* phylogroups and used for specificity evaluations: K-12 MG1655, 97054-1 (O119:NM), and EC05-66 (O108:H40) from *E. coli* phylogroup A; SE11 (O152:H18), 11368 (O26:H11), and 12009 (O103:H2) from phylogroup B1; E2348/69 (O127:H6), SE15 (O150:H5), and EC04-268 (O21:H6) from phylogroup B2; 97604 (O167:HND) and 94414 (OUT:NM) from phylogroup D; and Sakai (O157:H7), 97105 (OUT:HND), and NIAH_Por_10 (O88:HND) from phylogroup E. Although no strains of *E. fergusonii* and *Escherichia* cryptic clades were tested, the target sequences of all primers were absent in the sequenced strains of these species and clades.
