# Supplementary material for: Defining the Genome Features of Escherichia albertii, an Emerging Enteropathogen Closely Related to Escherichia coli
Source: Genome Biol Evol. 2015 Nov 3;7(12):3170–9. doi: 10.1093/gbe/evv211 (PMC4700944; doi:10.1093/gbe/evv211)
Supplement: Supplementary Data [file supp_evv211_Supplemental_Figures_GBEre.pdf]

Fig.S1

A

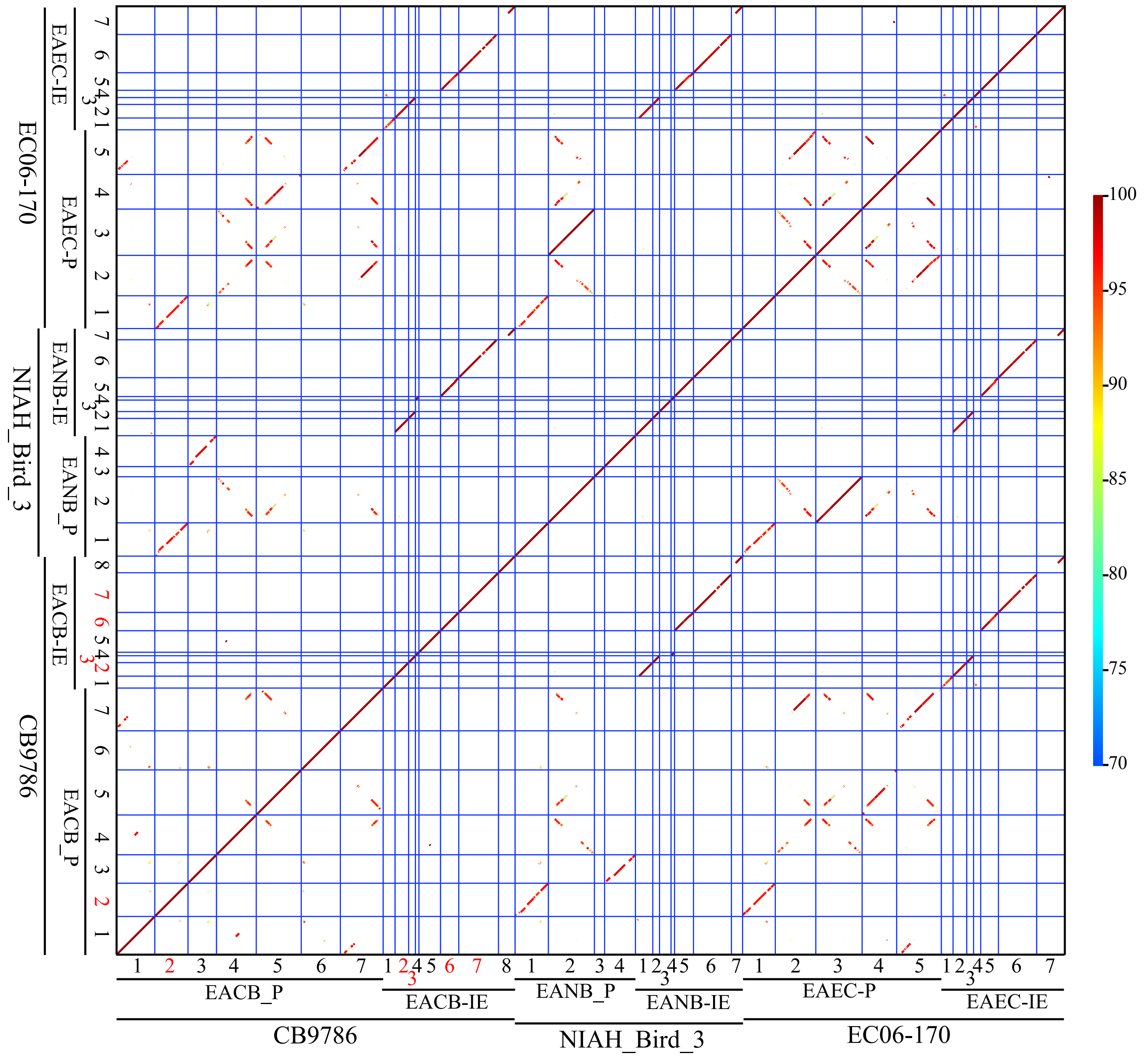

EC06-170
EAEC-IE
EAEC-P
NIAH_Bird_3
EANB-IE
EANB_P
CB9786
EACB-IE
EACB_P
EACB_P
EACB-IE
CB9786
EANB_P
EANB-IE
NIAH_Bird_3
EAEC-P
EAEC-IE
EC06-170
100
95
90
85
80
75
70

B

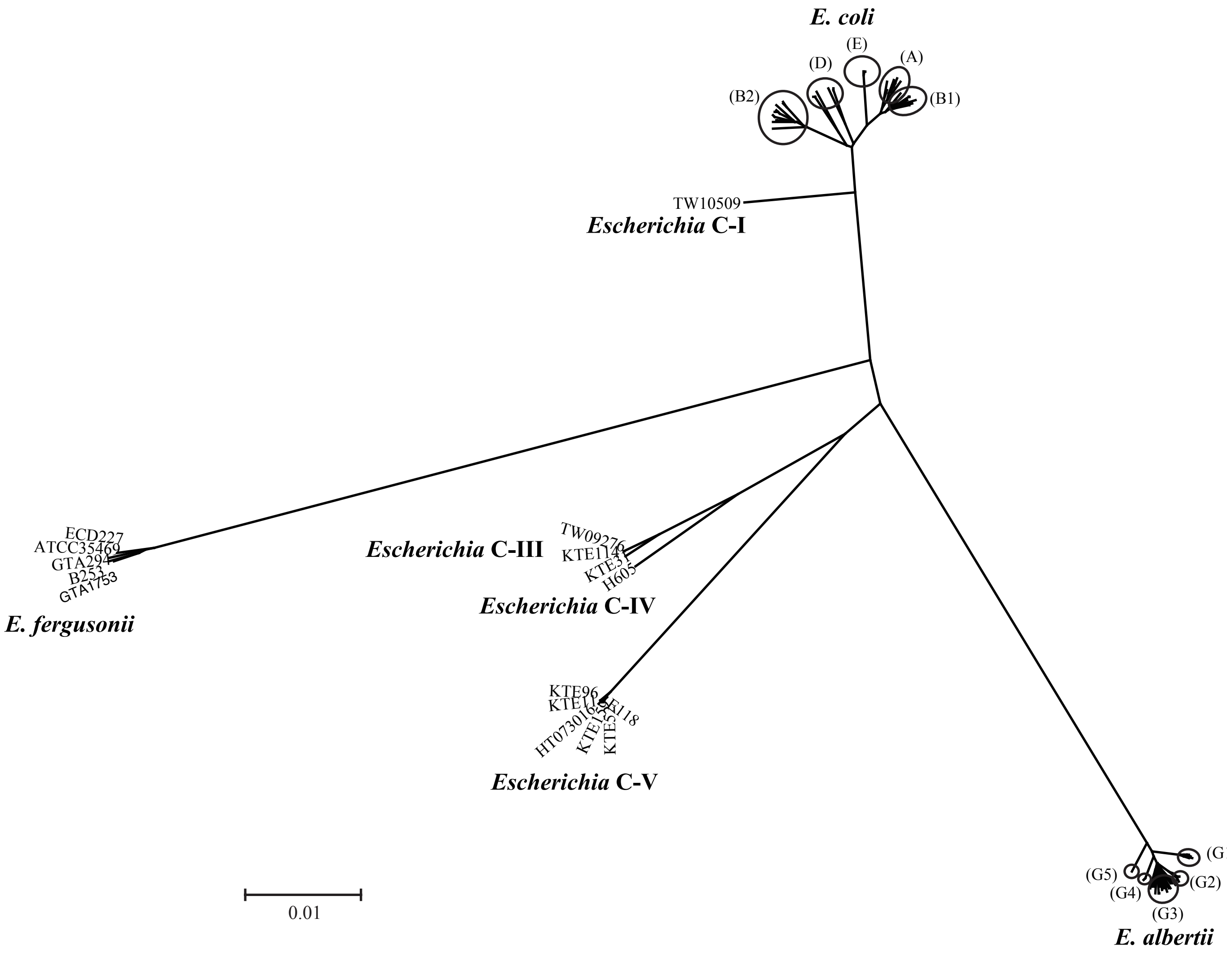
E. coli
(E)
(D)
(A)
(B2)
(B1)
TW10509
Escherichia C-I
ECD227
ATCC35469
B253
GTA1753
E. fergusonii
Escherichia C-III
TW09276
Escherichia C-IV
H605
KTE96
Escherichia C-V
(G1)
(G5)
(G2)
(G4)
(G3)
E. albertii
0.01

C

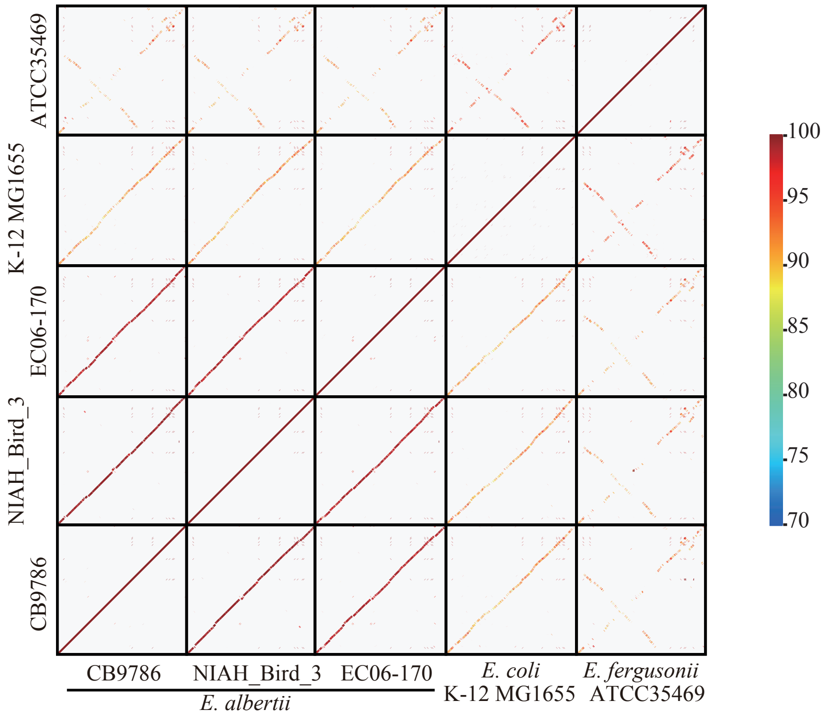

D

w/oPP&IE

whole

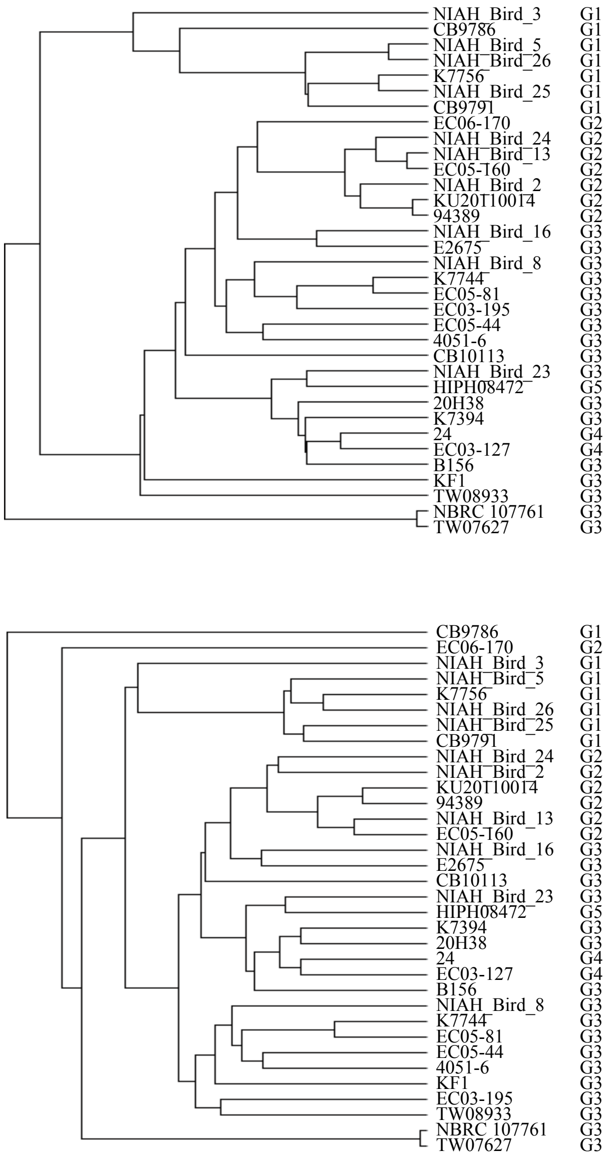

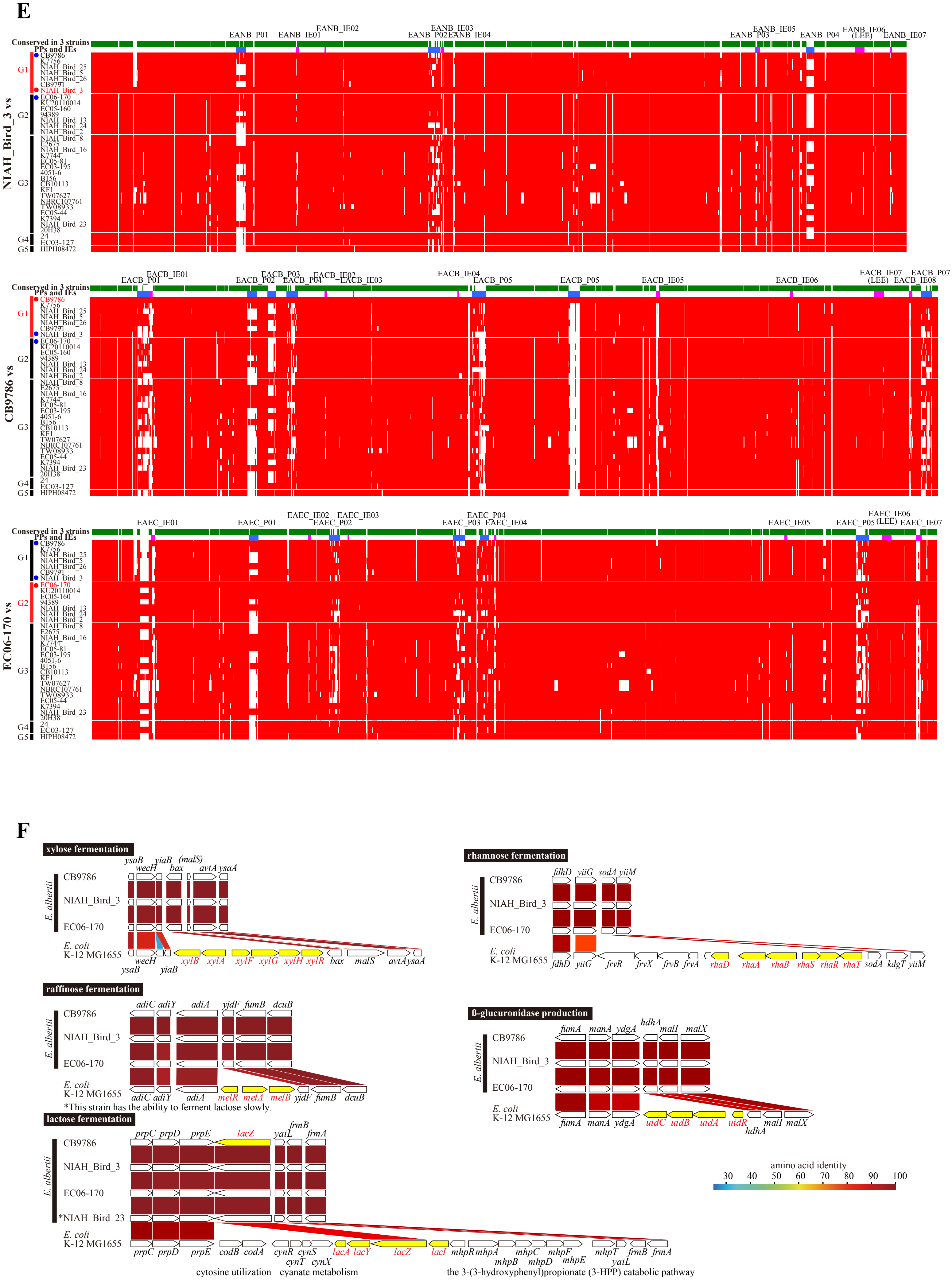
E
NIAH_Bird_3 vs
CB9786 vs
EC06-170 vs
Conserved in 3 strains
PPs and IEs
EANB_P01 EANB_IE01 EANB_IE02 EANB_IE03 EANB_P02 EANB_IE04 EANB_IE05 EANB_P03 EANB_P04 EANB_IE06 (LEE) EANB_IE07
EACB_IE01 EACB_P01 EACB_P03 EACB_P02 EACB_P04 EACB_IE02 EACB_IE03 EACB_IE04 EACB_P05 EACB_P05 EACB_IE05 EACB_IE06 EACB_IE07 (LEE) EACB_P07 EACB_IE08
EAEC_IE01 EAEC_P01 EAEC_IE02 EAEC_IE03 EAEC_P02 EAEC_P04 EAEC_P03 EAEC_IE04 EAEC_IE05 EAEC_IE06 EAEC_P05 (LEE) EAEC_IE07
G1 G2 G3 G4 G5
CB9786
K7756
NIAH_Bird_25
NIAH_Bird_5
NIAH_Bird_26
CB9791
NIAH_Bird_3
EC06-170
KU20110014
EC05-160
94389
NIAH_Bird_13
NIAH_Bird_24
NIAH_Bird_2
NIAH_Bird_8
E2675
NIAH_Bird_16
K7744
EC05-81
EC03-195
4051-6
B156
CB10113
KF1
TW07627
NBRC107761
TW08933
EC05-44
K7394
NIAH_Bird_23
20H38
24
EC03-127
HIPH08472
F
xylose fermentation
E. albertii
CB9786
NIAH_Bird_3
EC06-170
E. coli
K-12 MG1655
ysaB wecH yiaB bax (malS) avtA ysaA
xylB xylA xylF xylG xylH xylR bax malS avtA ysaA
rhamnose fermentation
fdhD yiiG sodA yiiM
fdhD yiiG frvR frvX frvB frvA rhaD rhaA rhaB rhaS rhaR rhaT sodA kdgT yiiM
raffinose fermentation
adiC adiY adiA yjdF fumB dcuB
adiC adiY adiA melR melA melB yjdF fumB dcuB
*This strain has the ability to ferment lactose slowly.
ß-glucuronidase production
fumA manA ydgA hdhA malI malX
fumA manA ydgA uidC uidB uidA uidR malI malX hdhA
lactose fermentation
*NIAH_Bird_23
prpC prpD prpE lacZ yaiL frmB frmA
prpC prpD prpE codB codA cynR cynS cynT cynX lacA lacY lacZ lacI mhpR mhpA mhpB mhpC mhpD mhpF mhpE mhpT yaiL frmB frmA
cytosine utilization
cyanate metabolism
the 3-(3-hydroxyphenyl)propionate (3-HPP) catabolic pathway
amino acid identity
30 40 50 60 70 80 90 100

Fig. S2

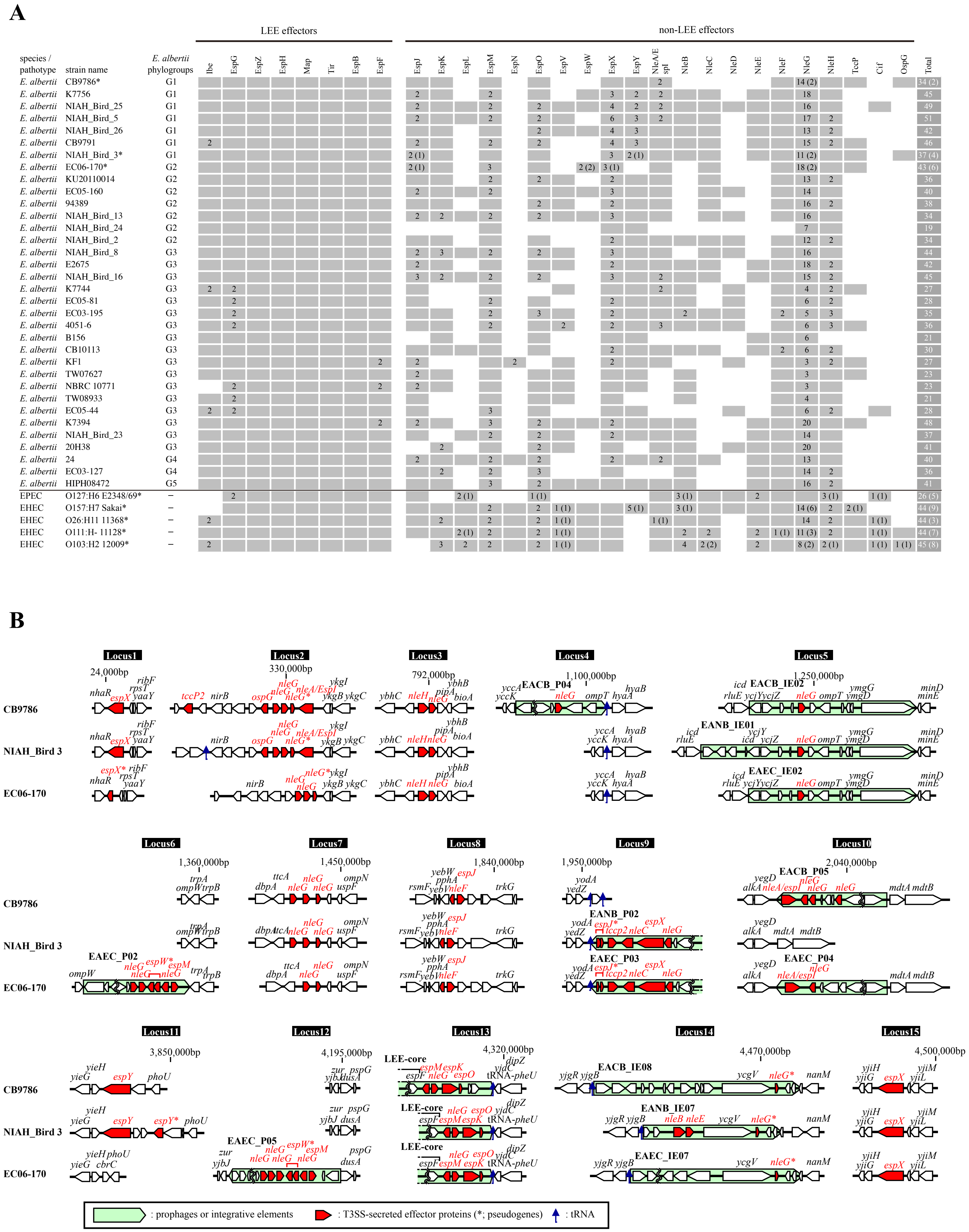
A
LEE effectors
non-LEE effectors
species / pathotype
strain name
E. albertii phylogroups
Ibe
EspG
EspZ
EspH
Map
Tir
EspB
EspF
EspJ
EspK
EspL
EspM
EspN
EspO
EspV
EspW
EspX
EspY
NleA/EspI
NleB
NleC
NleD
NleE
NleF
NleG
NleH
TccP
Cif
OspG
Total
E. albertii CB9786* G1
E. albertii K7756 G1
E. albertii NIAH_Bird_25 G1
E. albertii NIAH_Bird_5 G1
E. albertii NIAH_Bird_26 G1
E. albertii CB9791 G1
E. albertii NIAH_Bird_3* G1
E. albertii EC06-170* G2
E. albertii KU20110014 G2
E. albertii EC05-160 G2
E. albertii 94389 G2
E. albertii NIAH_Bird_13 G2
E. albertii NIAH_Bird_24 G2
E. albertii NIAH_Bird_2 G2
E. albertii NIAH_Bird_8 G3
E. albertii E2675 G3
E. albertii NIAH_Bird_16 G3
E. albertii K7744 G3
E. albertii EC05-81 G3
E. albertii EC03-195 G3
E. albertii 4051-6 G3
E. albertii B156 G3
E. albertii CB10113 G3
E. albertii KF1 G3
E. albertii TW07627 G3
E. albertii NBRC 10771 G3
E. albertii TW08933 G3
E. albertii EC05-44 G3
E. albertii K7394 G3
E. albertii NIAH_Bird_23 G3
E. albertii 20H38 G3
E. albertii 24 G4
E. albertii EC03-127 G4
E. albertii HIPH08472 G5
EPEC O127:H6 E2348/69* –
EHEC O157:H7 Sakai* –
EHEC O26:H11 11368* –
EHEC O111:H- 11128* –
EHEC O103:H2 12009* –
B
Locus1
Locus2
Locus3
Locus4
Locus5
Locus6
Locus7
Locus8
Locus9
Locus10
Locus11
Locus12
Locus13
Locus14
Locus15
CB9786
NIAH_Bird 3
EC06-170
EACB_P04
EACB_IE02
EANB_IE01
EAEC_IE02
EAEC_P02
EACB_P05
EANB_P02
EAEC_P03
EAEC_P04
EAEC_P05
LEE-core
EACB_IE08
EANB_IE07
EAEC_IE07
: prophages or integrative elements
: T3SS-secreted effector proteins (*; pseudogenes)
: tRNA

Fig. S3

A

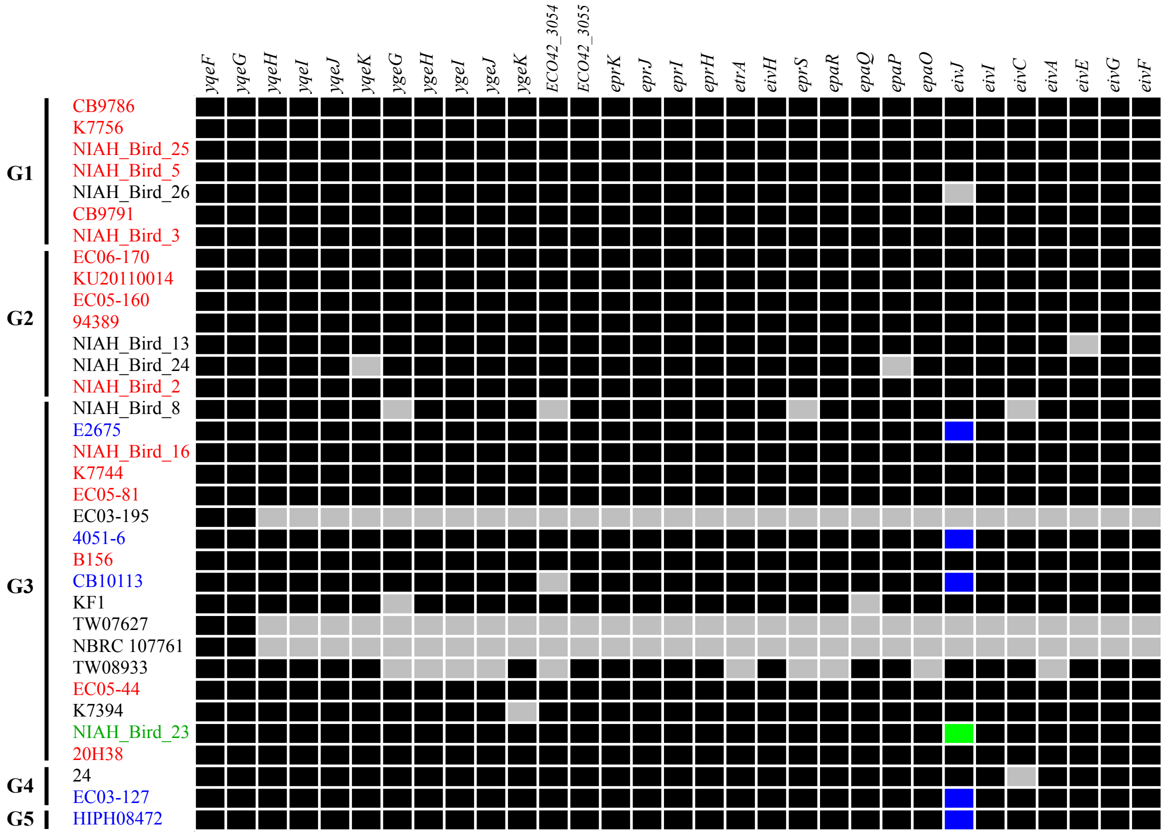

B

```
                       T  E  K  V  N  N  T  V  N  Y  L  I  K  K  N   A  N  K  S  V  V  K  A  D  K  S  E  V  N  E
CB9786(intact) 325 ACTGAAAAGGTTAATAATACCGTAAATTATTTGATTAAAAAAAA-TGCAAATAAATCTGTAGTAAAAGCTGATAAATCAGAAGTAAATGAA 415
E2675          325 ACTGAAAAGGTTAATAATACCGTAAATTATTTGATTAAAAAAA--TGCAAATAAATCTGTAGTAAAAGCTGATAAATCAGAAGTAAATGAA 414
CB10113        325 ACTGAAAAGGTTAATAATACCGTAAATTATTTGATTAAAAAAA--TGCAAATAAATCTGTAGTAAAAGCTGATAAATCAGAAGTAAATGAA 414
4051-6         325 ACTGAAAAGGTTAATAATACCGTAAATTATTTGATTAAAAAAA--TGCAAATAAATCTGTAGTAAAAGCTGATAAATCAGAAGTAAATGAA 414
NIAH_Bird_23   325 ACTGAAAAGGTTAATAATACCGTAAATTATTTGATTAAAAAAAAATGCAAATAAATCTGTAGTAAAAGCTGATAAATTAGAAGTAAATGAA 416
EC03-127       325 ACTGAAAAGGTTAATAATACCGTAAATTATTTGATTAAAAAAA--TGCAAATAAATCTGTAGTAAAAGCTGATAAATCAGAAGTAAATGAA 414
HIPH08472      325 ACTGAAAAGGTTAATAATACCGTAAATTATTTGATTAAAAAAA--TGCAAATAAATCTGTAGTAAAAGCTGATAAATCAGAAGTAAATGAA 414
                   *******************************************  ********************************.************
```

C

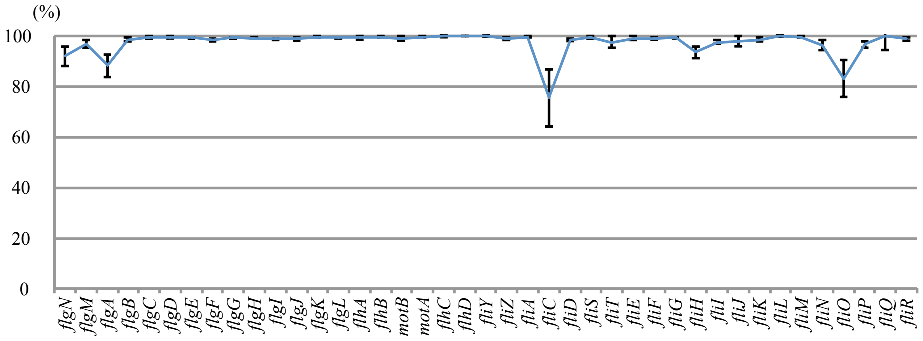

D

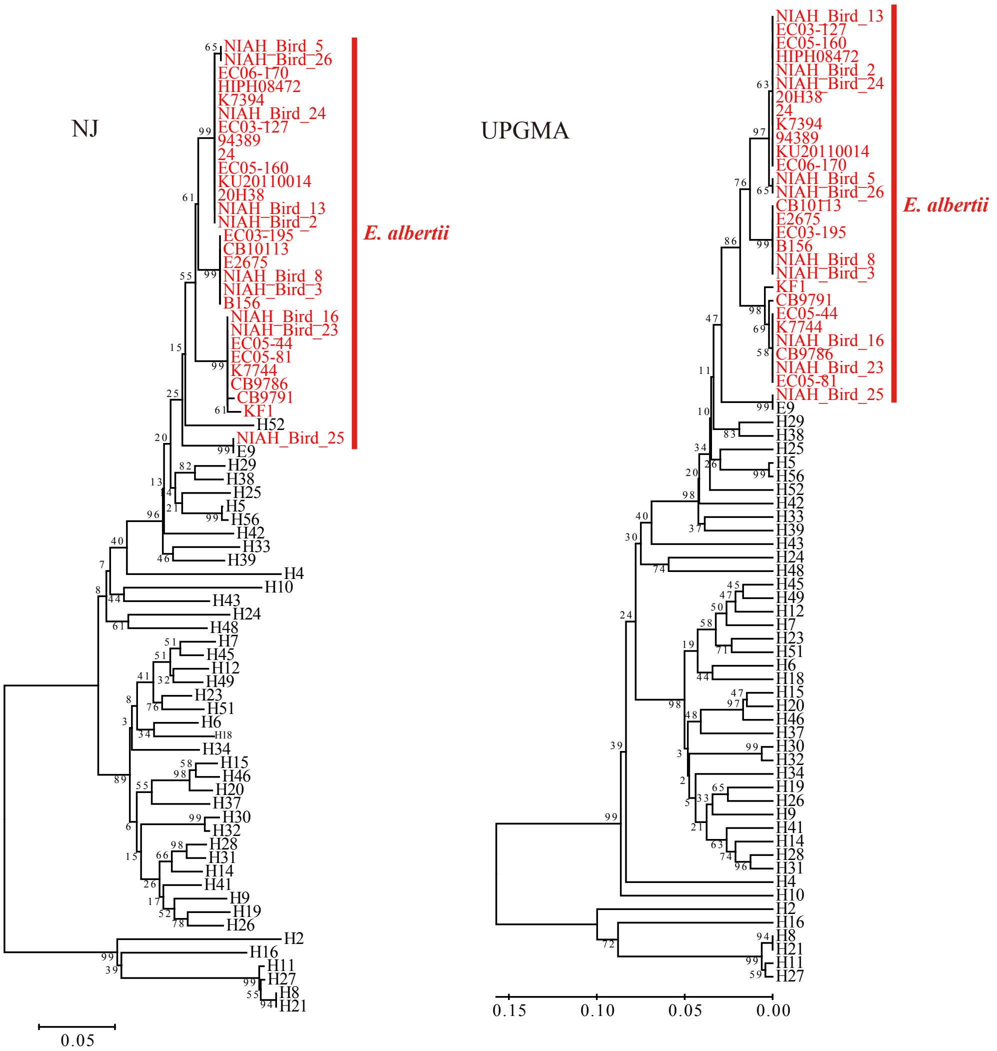
NJ
UPGMA
E. albertii
NIAH_Bird_5
NIAH_Bird_26
EC06-170
HIPH08472
K7394
NIAH_Bird_24
EC03-127
94389
24
EC05-160
KU20110014
20H38
NIAH_Bird_13
NIAH_Bird_2
EC03-195
CB10113
E2675
NIAH_Bird_8
NIAH_Bird_3
B156
NIAH_Bird_16
NIAH_Bird_23
EC05-44
EC05-81
K7744
CB9786
CB9791
KF1
H52
NIAH_Bird_25
E9
0.05
0.15
0.10
0.05
0.00

E

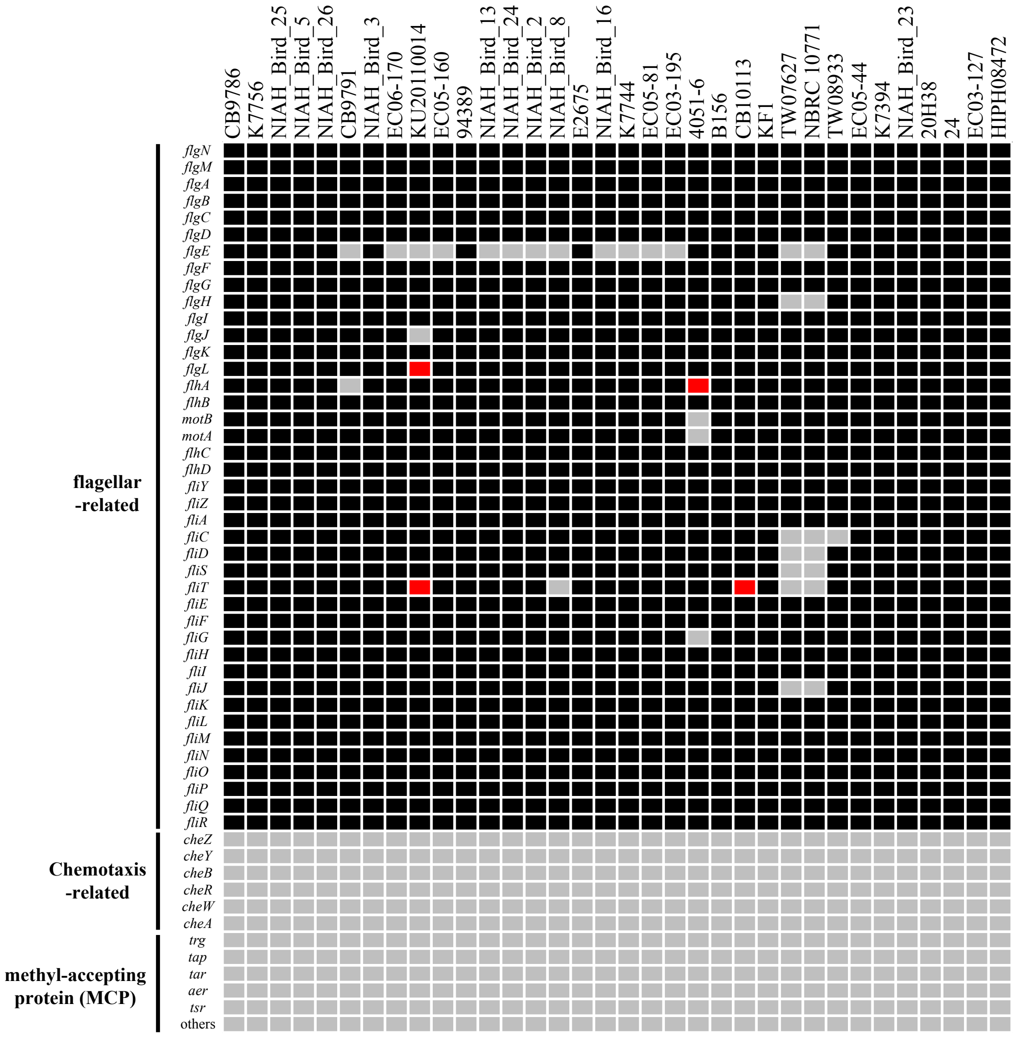
CB9786
K7756
NIAH_Bird_25
NIAH_Bird_5
NIAH_Bird_26
CB9791
NIAH_Bird_3
EC06-170
KU20110014
EC05-160
94389
NIAH_Bird_13
NIAH_Bird_24
NIAH_Bird_2
NIAH_Bird_8
E2675
NIAH_Bird_16
K7744
EC05-81
EC03-195
4051-6
B156
CB10113
KF1
TW07627
NBRC 10771
TW08933
EC05-44
K7394
NIAH_Bird_23
20H38
24
EC03-127
HIPH08472
flagellar -related
flgN
flgM
flgA
flgB
flgC
flgD
flgE
flgF
flgG
flgH
flgI
flgJ
flgK
flgL
flhA
flhB
motB
motA
flhC
flhD
fliY
fliZ
fliA
fliC
fliD
fliS
fliT
fliE
fliF
fliG
fliH
fliI
fliJ
fliK
fliL
fliM
fliN
fliO
fliP
fliQ
fliR
Chemotaxis -related
cheZ
cheY
cheB
cheR
cheW
cheA
methyl-accepting protein (MCP)
trg
tap
tar
aer
tsr
others

F

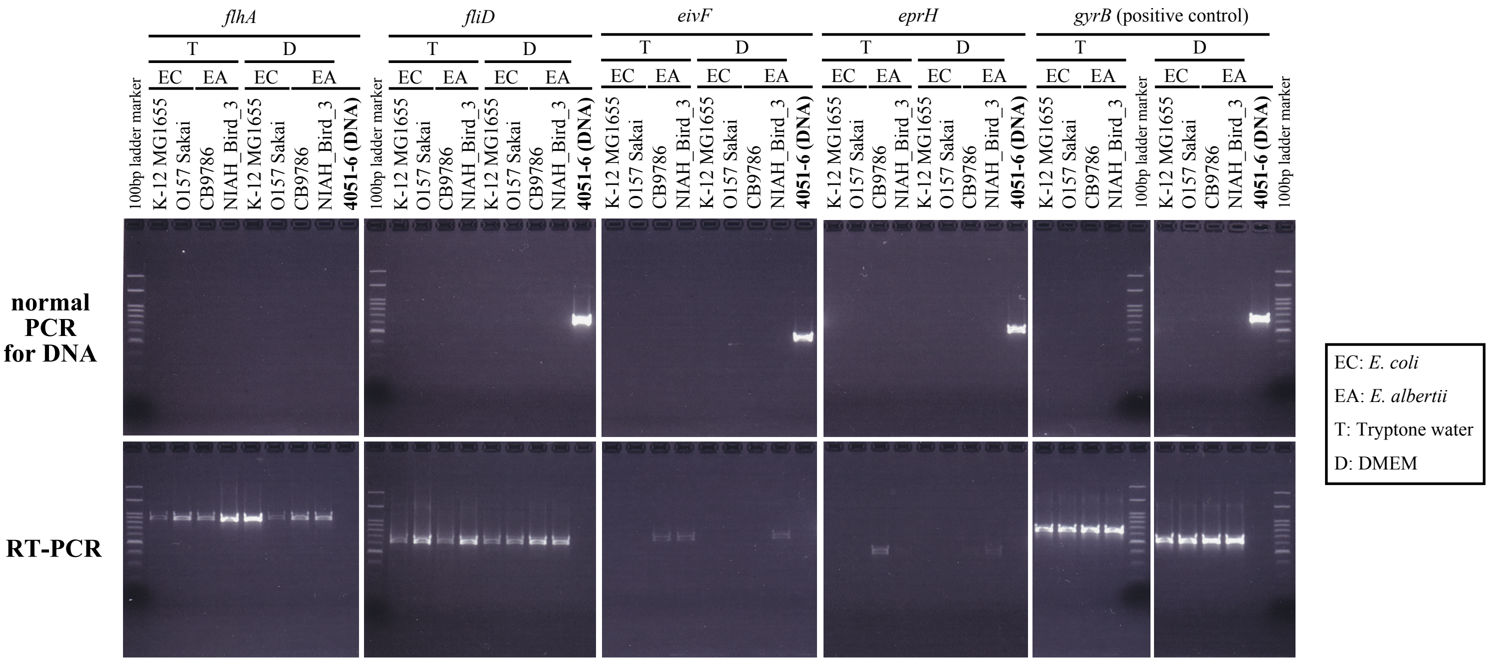

flhA
fliD
eivF
eprH
gyrB (positive control)
T
D
EC
EA
100bp ladder marker
K-12 MG1655
O157 Sakai
CB9786
NIAH_Bird_3
4051-6 (DNA)
normal PCR for DNA
RT-PCR
EC: E. coli
EA: E. albertii
T: Tryptone water
D: DMEM

Fig. S4

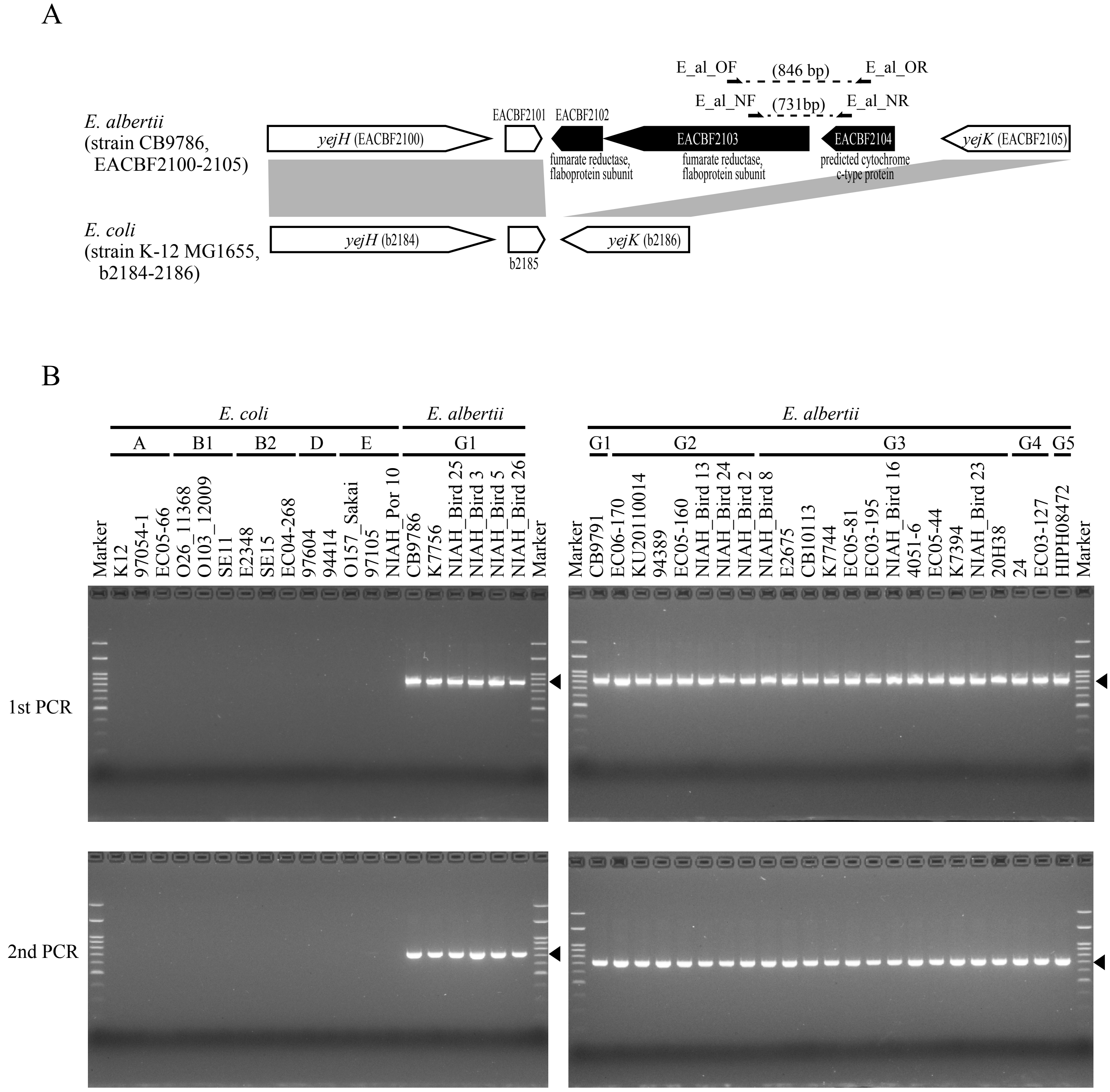
A
E_al_OF
(846 bp)
E_al_OR
E_al_NF
(731bp)
E_al_NR
E. albertii (strain CB9786, EACBF2100-2105)
yejH (EACBF2100)
EACBF2101
EACBF2102
EACBF2103
EACBF2104
yejK (EACBF2105)
fumarate reductase, flaboprotein subunit
fumarate reductase, flaboprotein subunit
predicted cytochrome c-type protein
E. coli (strain K-12 MG1655, b2184-2186)
yejH (b2184)
b2185
yejK (b2186)
B
E. coli
E. albertii
A
B1
B2
D
E
G1
G2
G3
G4
G5
Marker
K12
97054-1
EC05-66
O26_11368
O103_12009
SE11
E2348
SE15
EC04-268
97604
94414
O157_Sakai
97105
NIAH_Por 10
CB9786
K7756
NIAH_Bird 25
NIAH_Bird 3
NIAH_Bird 5
NIAH_Bird 26
CB9791
EC06-170
KU20110014
94389
EC05-160
NIAH_Bird 13
NIAH_Bird 24
NIAH_Bird 2
NIAH_Bird 8
E2675
CB10113
K7744
EC05-81
EC03-195
NIAH_Bird 16
4051-6
EC05-44
K7394
NIAH_Bird 23
20H38
24
EC03-127
HIPH08472
1st PCR
2nd PCR
