# Supplementary material for: Defining the Genome Features of Escherichia albertii, an Emerging Enteropathogen Closely Related to Escherichia coli
Source: Genome Biol Evol. 2015 Nov 3;7(12):3170–9. doi: 10.1093/gbe/evv211 (PMC4700944; doi:10.1093/gbe/evv211)
Supplement: Supplementary Data [file supp_evv211_Supplemental_Tables_GBEre.pdf]

Table S1. The list of *E. albertii* strains sequenced in this study and the summary of genome sequencing

strain name	Phylogroup of *E. albertii*	sequencing status[a]	total number of reads	>500 bp scaffolds	sequence redundancy	N50 scaffold length (bp)	longest scaffold length (bp)	Genome size or total length of >500 bp scaffolds (bp)	year of isolation	source[b]	contries[c]	intimin subtype	notes	References	accession No.
CB9786	G1	C	–	–	–	–	–	4,598,983	2003	Human, symptomatic (d)	G	alpha8		(1)	AP014856
NIAH_Bird_3	G1	C	–	–	–	–	–	4,560,575	2004	Bird (*Puffinus tenuirostris*)	J	N1.1		(1)	AP014855
EC06-170	G2	C	–	–	–	–	–	4,657,167	2006	Human, symptomatic (NI)	J	xi		(1)	AP014857
K7756	G1	D	4,448,778	53	120	262,646	547,062	4,727,433	2009	Human, symptomatic (NI)	J	sigma		this study	BBWC01000001-BBWC01000053
NIAH_Bird_25	G1	D	1,528,568	113	38	167,130	341,925	4,776,752	2006	Bird (*Passer montanus*)	J	sigma		(1)	BBVN01000001-BBVN01000113
NIAH_Bird_5	G1	D	3,172,674	106	80	144,618	383,621	4,830,654	2004	Bird (*Puffinus tenuirostris*)	J	beta3		(1)	BBVP01000001-BBVP01000106
NIAH_Bird_26	G1	D	1,708,964	103	52	240,307	585,919	4,710,100	2006	Bird (*Hypsipetes amaurotis*)	J	beta3		(1)	BBVO01000001-BBVO01000103
CB9791	G1	D	1,760,488	100	52	167,119	441,440	4,760,418	2003	Human, symptomatic (d)	G	alpha8		(1)	BBVS01000001-BBVS01000100
KU20110014	G2	D	1,764,634	111	50	117,550	382,132	4,780,591	2011	H (S [d, ap, f])	J	sigma	outbreak	(2)	BBWD01000001-BBWD01000111
94389	G2	D	1,461,990	112	39	113,364	258,750	4,745,744	1994	Human, NI	J	sigma		(1)	BBVH01000001-BBVH01000112
EC05-160	G2	D	1,755,200	73	57	213,245	529,418	4,739,675	2005	Human, symptomatic (NI)	J	sigma		(1)	BBVW01000001-BBVW01000073
NIAH_Bird_13	G2	D	2,617,298	63	70	219,808	460,906	4,726,449	2005	Bird (*Hypsipetes amaurotis*)	J	xi		(1)	BBVI01000001-BBVI01000063
NIAH_Bird_24	G2	D	1,859,030	94	55	182,032	438,619	4,783,301	2006	Bird (*Cyanopica cyana*)	J	sigma		(1)	BBVM01000001-BBVM01000094
NIAH_Bird_2	G2	D	2,608,708	70	80	173,541	498,643	4,511,428	2002	Bird (*Sturnus cineraceus*)	J	sigma		(1)	BBVK01000001-BBVK01000070
NIAH_Bird_8	G3	D	2,301,508	167	65	73,046	218,040	4,770,174	2004	Bird (*Egretta garzetta*)	J	epsilon4		(1)	BBVQ01000001-BBVQ01000167
E2675	G3	D	2,242,944	119	50	139,498	378,776	4,958,535	2007	Bird (Corvus)	J	N1.2	*stx2f* (+)	(1)	BBVT01000001-BBVT01000119
CB10113	G3	D	1,994,412	144	53	124,577	370,016	4,715,204	2004	Cat (AC)	B	ypsilon		(1)	BBVR01000001-BBVR01000144
K7744	G3	D	2,711,886	116	67	132,486	206,201	4,844,216	2009	Human, symptomatic (NI)	J	jota		this study	BBWB01000001-BBWB01000116
EC05-81	G3	D	1,803,922	50	55	221,444	515,879	4,656,922	2005	Human, symptomatic (NI)	J	N3		(1)	BBVY01000001-BBVY01000050
EC03-195	G3	D	1,774,536	78	52	285,050	437,317	4,853,676	2003	Human, symptomatic (NI)	J	N5		(1)	BBVV01000001-BBVV01000078
NIAH_Bird_16	G3	D	1,702,536	145	45	102,938	380,167	5,013,639	2005	Bird (*Passer montanus*)	J	N1.1		(1)	BBVJ01000001-BBVJ01000145
4051-6	G3	D	1,525,534	84	100	208980	346,931	4,700,514	1989	Human, symptomatic (d)	B	omicron		(1)	BBVG01000001-BBVG01000084
EC05-44	G3	D	1,663,840	43	57	265,550	608,443	4,571,045	2005	Human, symptomatic (NI)	J	N4		(1)	BBVX01000001-BBVX01000043
K7394	G3	D	3,263,926	59	77	518,299	353,317	5,120,257	2008	Human, symptomatic (NI)	J	sigma		this study	BBWA01000001-BBWA01000059
NIAH_Bird_23	G3	D	1,819,260	102	52	157,590	397,573	4,796,871	2006	Bird (*Phalacrocorax carbo*)	J	epsilon1		(1)	BBVL01000001-BBVL01000102
20H38	G3	D	1,514,890	92	41	154,251	346,678	4,862,440	2008	Human, symptomatic (d)	J	sigma		(1)	BBVE01000001-BBVE01000092
24	G4	D	10,309,770	137	224	199,156	315,633	4,898,975	2000	Human, symptomatic (d, ap)	J	(epsilon3)		(1)	BBVF01000001-BBVF01000137
EC03-127	G4	D	1,984,408	149	45	136,933	339,841	4,997,600	2003	Human, symptomatic (d)	J	(epsilon3)		(1)	BBVU01000001-BBVU01000149
HIPH08472	G5	D	9,589,256	85	235	294,711	686,091	5,028,180	2008	Human, symptomatic (d)	J	N2	*stx2f* (+)	(1)	BBVZ01000001-BBVZ01000085

Ref. 1; Ooka T, et al. (2012) Clinical significance of *Escherichia albertii*. Emerg Infect Dis 18(3):488-492.

Ref. 2; Ooka T, et al. (2013) Human gastroenteritis outbreak associated with *Escherichia albertii*, Japan. Emerg Infect Dis 19(1):144-146.

[a] C: complete, D: draft

[b] Symptoms of the patients, from which each clinical strain was isolated, are indicated in parentheses (d: diarrhea, ap: abdominal pain, f: fever, NI: no information).

[c] G: Germany, B: Brazil, J: Japan

Table S2. *E. coli*, *E. fergusonii* and *E. albertii* strains used for comparative genome analysis

Completely sequenced strains

Strain	Phylogroup	Pathotype[b]	Serotype[b]	Chromosome				Plasmids			total numbers of CDSs	Reference
				Size (kb)	CDSs[a]	tRNA	Accession number	Size (kb)	CDSs[a]	Accession number		
O55_CB9615[c]	E	EHEC	O55:H7	5,386	5,014	100	CP001846	66	107	CP001847	5,121	(1)
O55_RM12579	E	EHEC	O55:H7	5,264	4,912	102	CP003109	94/66/12/6/6	216	CP003110-14	5,128	(2)
O157_Sakai[c]	E	EHEC	O157:H7	5,498	5,363 (247)	105	BA000007	93/3	95 (11)	AB011548-9	4,807	(3)
O157_TW14359	E	EHEC	O157:H7	5,528	5,263	106	CP001368	95	110	CP001369	5,373	(4)
O157_EDL933	E	EHEC	O157:H7	5,528	5,349	98	AE005174	92	100	AF074613	5,449	(5)
O157_EC4115	E	EHEC	O157:H7	5,572	5,315	110	CP001164	95/37	162	CP001163, 11655	5,477	(6)
O157_Xuzhou21	E	EHEC	O157:H7	5,386	5,039	93	CP001925	92/38	144	CP001926-7	5,183	(7)
UMNK88	A	porcine ETEC	NI	5,186	5,118	96	CP002729	161/91/82/81/66	490	CP002730-33, HQ023862	5,608	unpublished
H10407	A	ETEC	O78	5,153	4,763	89	FN649414	95/67/6/5/5	249	FN649415-8	5,012	(16)
MG1655[c]	A	laboratory	-	4,639	4,294 (101)	88	U00096	-	-	-	4,033	(26)
P12b	A	NI	NI	4,935	4,393	83	CP002291	-	-	-	4,393	(17)
ATCC8739	A	NI	NI	4,746	4,200	87	CP000946	-	-	-	4,200	unpublished
HS[c]	A	commensal	O9	4,643	4,478 (94)	88	CP000802	-	-	-	4,248	(10)
APECO78	B1	APEC	O78	4,798	4,589	88	CP004009	-	-	-	4,589	unpublished
O26_11368[c]	B1	EHEC	O26:H11	5,697	5,609 (254)	101	AP010953	85/63/6/4	186 (31)	AP010954-7	4,946	(8)
O111_11128	B1	EHEC	O111:H-	5,371	5,264 (291)	106	AP010960	205/98/78/8/7	468 (33)	AP010961-5	4,965	(8)
O103_12009	B1	EHEC	O103:H2	5,449	5,264	98	AP010958	72	90	AP010959	5,336	(8)
IAI1	B1	commensal	O8	4,701	4,491 (51)	94	CU928160	-	-	-	4,267	(25)
SE11[c]	B1	commensal	O152:H28	4,888	4679	86	AP009240	100/91/61/7/5/4	323	AP009241-6	4,821	(11)
2009EL-2050	B1	EAEC/EHEC	O104:H4	5,253	4,970	95	CP003297	109/74/2	213	CP003298-3300	5,183	(20)
2009EL-2071	B1	EAEC/EHEC	O104:H4	5,313	5,040	99	CP003301	76/2	86	CP003302-3	5,126	(20)
2011C-3493	B1	EAEC/EHEC	O104:H4	5,273	4,975	94	CP003289	89/74/2	175	CP003290-2	5,150	(20)
55989	B1	EAEC	O104:H4	5,155	4,969 (79)	86	CU928145	72	100 (7)	CU928159	4,635	(25)
KO11FL	B1	laboratory	NI	5,021	4,697	85	CP002970	5	12	CP002971	4,709	(23)
E24377A	B1	ETEC	O139:H28	4,979	4,873 (118)	91	CP000800	79/74/70/34/6/5	242 (34)	CP000795-9, 801	4,760	(10)
042[c]	D	EAEC	O44:H18	5,242	4,810	93	FN554766	113	152	FN554767	4,962	(14)
UMN026[c]	D	ExPEC	O17:K52:H18	5,202	4,918 (45)	88	CU928163	122/34	198 (8)	CU928148-9	4,867	(25)
IAI39	D	ExPEC	O7:K1	5,132	4,906 (80)	88	CU928164	-	-	-	4,432	(25)
CE10	D	ExPEC	O7:K1:NM	5,314	5,009	91	CP003034	54/5/4/1	71	CP003035-8	5,080	(9)
SMS-3-5	D	environment	O19:H34	5,068	4,743	90	CP000970	130/9/4/3	170	CP000971-4	4,913	(13)
E2348/69[c]	B2	EPEC	O127:H6	4,965	4,703 (145)	92	FM180568	97/6	121 (23)	FM180569-70	4,497	(27)
536	B2	ExPEC	O6:K15:H7	4,938	4,685	81	CP000247	-	-	-	4,457	(29)
NRG857C	B2	AIEC	O83:H1	4,748	4,429	84	CP001855	147	153	CP001856	4,582	(15)
LF82	B2	AIEC	NI	4,773	4,376	84	CU651637	-	-	-	4,376	unpublished
ABU_83972	B2	commensal	NI	5,131	4,793	88	CP001671	2	3	CP001833	4,795	(24)
CFT073	B2	ExPEC	O6:K2:H1	5,231	5,473 (94)	89	AE14075	-	-	-	5,101	(28)
ED1a	B2	commensal	O81	5,210	5,129 (95)	91	CU928162	120	150 (11)	CU928147	4,634	(25)
UM146	B2	AIEC	NI	4,993	4,650	86	CP002167	115	133	CP002168	4,783	(22)
UTI89	B2	ExPEC	-	5,065	5,066	88	CP000243	114	145	CP000244	5,073	(30)
IHE3034	B1	ExPEC	O18:K1:H7	5,108	4,757	97	CP001969	-	-	-	4,757	(21)
APEC O1	B2	ExPEC	O1:K1:H7	5,082	4,467	94	CP000468	241/174	423	DQ517526, DQ381420	4,730	(19)
S88	B2	ExPEC	O45:K1:H7	5,032	4,859 (90)	91	CU928161	134	144 (9)	CU928146	4,666	(25)
SE15[c]	B2	commensal	O150:H5	4,717	4,338	84	AP009378	122	150	AP009379	4,488	(12)
NA114	B2	ExPEC	O25	4,971	4,873	79	CP002797	-	-	-	4,873	(18)
ATCC35469[c]	*E. fergusonii*		-	4,655	4,319	87	CU928158	55	58	CU928144	4,377	unpublished
KF1	*E. albertii*		NI	4,702	4,422	85	CP007025	-	-	-	-	(31)

Draft-sequences

Strain	Species or clades	Pathotype[b]	Total length (kb)	Sequening platform[b]	Sequence redundancy[b]	N50 length (bp)[b]	Accession number
NBRC 107761	*E. albertii*	diarrheic	4,422	Illumina	150	29,757	BBMY00000000
TW07627	*E. albertii*	diarrheic	4,747	Roche454	23	395,133	ABKX00000000
TW08933	*E. albertii*	NI	4,513	Illumina	600	27,943	AEJU00000000
B156	*E. albertii*	avirulent	4,996	NI	NI	NI	directly collect from Broad institute
TW10509[c]	CC-I	ETEC	5,191	Illumina	600	27,634	AEKA00000000
TW09276[c]	CC-III	avirulent	4,473	Illumina	600	28,680	AEJV00000000
H605[c]	CC-IV	avirulent	4,482	NI	NI	NI	directly collect from Broad institute
E1118[c]	CC-V	avirulent	4,617	NI	NI	NI	directly collect from Broad institute
KTE11[c]	CC-V	NI	4,527	Illumina	261	229,669	ANSR00000000
KTE96[c]	CC-V	NI (human)	4,608	Illumina	130	229,293	ASVD00000000
KTE114[c]	CC-III	NI	4,754	Illumina	142	515,979	ASTS00000000
KTE31[c]	CC-III	NI	4,557	Illumina	141	454,369	ASTZ00000000
KTE159[c]	CC-V	NI	4,810	Illumina	136	192,611	ASVR00000000
KTE52[c]	CC-V	NI	4,648	Illumina	135	157,654	ASUT00000000
HT073016[c]	CC-V / *E. marmotae*	NI	4,506	Illumina	100	44,977	JNBP00000000
[illegible]-4-RBA-P5[c]	*E. fergusonii*	NI	4,764	Illumina	31	268,310	JZWQ00000000
B253[c]	*E. fergusonii*	NI	4,776	Roche454	34	284,983	AEIA00000000
ECD227[c]	*E. fergusonii*	NI	4,866	Roche454	18	156,910	AEVY00000000
[illegible]-294-5-RBA-P2[c]	*E. fergusonii*	NI	4,495	Illumina	23	298,179	JZWN00000000

[a] Total numbers of CDSs including pseudogenes are shown. Numbers of pseudogenes are indicated in parentheses if available.

[b] NI: no information

[c] These strains were used for ANI analysis.

References are:

1. Zhou Z, et al. (2010) Derivation of *Escherichia coli* O157:H7 from its O55:H7 precursor. Plos ONE 5(1):E8700.
2. Kyle JL, et al. (2012) *Escherichia coli* Serotype O55:H7 Diversity Supports Parallel Acquisition of Bacteriophage at Shiga Toxin Phage Insertion Sites during Evolution of the O157:H7 Lineage. J Bacteriol 194(8):1885-1896.
3. Hayashi T, et al. (2001) Complete genome sequence of enterohemorrhagic *Escherichia coli* O157:H7 and genomic comparison with a laboratory strain K-12. DNA Res 8(1):11-22.
4. Kulasekara BR, et al. (2009) Analysis of the genome of the *Escherichia coli* O157:H7 2006 spinach-associated outbreak isolate indicates candidate genes that may enhance virulence. Infect Immun 77(9):3713-3721.
5. Perna NT, et al. (2001) Genome sequence of enterohaemorrhagic *Escherichia coli* O157:H7. Nature 409(6819):529-533.
6. Eppinger M, et al. (2011) Genomic anatomy of *Escherichia coli* O157:H7 outbreaks. Proc Natl Acad Sci U S A 108(50):20142-20147.
7. Xiong Y, et al. (2012) A Novel *Escherichia coli* O157:H7 Clone Causing a Major Hemolytic Uremic Syndrome Outbreak in China. Plos ONE 7(4):E36144.
8. Ogura Y, et al. (2009) Comparative genomics reveal the mechanism of the parallel evolution of O157 and non-O157 enterohemorrhagic *Escherichia coli*. Proc Natl Acad Sci U S A 106 (42):17939-17944.
9. Lu S, et al. (2011) Complete Genome Sequence of the Neonatal-Meningitis-Associated *Escherichia coli* Strain CE10. J Bacteriol 193(24):7005.
10. Rasko DA, et al. (2008) The pangenome structure of *Escherichia coli*: comparative genomic analysis of E. coli commensal and pathogenic isolates. J Bacteriol 190(20):6881-6893.
11. Oshima K, et al. (2008) Complete genome sequence and comparative analysis of the wild-type commensal *Escherichia coli* strain SE11 isolated from a healthy adult. DNA Res 15(6):375-386.
12. Toh H. (2010) Complete genome sequence of the wild-type commensal *Escherichia coli* strain SE15, belonging to phylogenetic group B2. J Bacteriol 192(4):1165-1166.
13. Fricke WF, et al. (2008) Insights into the environmental resistance gene pool from the genome sequence of the multidrug-resistant environmental isolate *Escherichia coli* SMS-3-5. J Bacteriol 190(20):6779-6794.
14. Chaudhuri RR, et al. (2010) Complete Genome Sequence and Comparative Metabolic Profiling of the Prototypical Enteroaggregative *Escherichia coli* Strain 042. Plos One 5(1):e8801.
15. Nash JH, et al. (2010) Genome sequence of adherent-invasive *Escherichia coli* and comparative genomic analysis with other *E. coli* pathotypes. BMC Genomics 11(1):667.
16. Crossman LC, et al. (2010) A commensal gone bad: complete genome sequence of the prototypical enterotoxigenic *Escherichia coli* strain H10407. J Bacteriol 192(21):5822-5831.

17. Liu B, et al. (2012) A novel non-homologous recombination-mediated mechanism for *Escherichia coli* unilateral flagellar phase variation. Nucleic Acids Res 40(10):4530-4538.
18. Avasthi TS, et al. (2011) Genome of Multidrug-Resistant Uropathogenic *Escherichia coli* Strain NA114 from India. J Bacteriol 193(16):4272-4273.
19. Johnson TJ, et al. (2007) The genome sequence of avian pathogenic *Escherichia coli* strain O1:K1:H7 shares strong similarities with human extraintestinal pathogenic *E. coli* genomes. J Bacteriol 189(8):3228-3236.
20. Ahmed SA, et al. (2012) Genomic Comparison of *Escherichia coli* O104:H4 Isolates from 2009 and 2011 Reveals Plasmid, and Prophage Heterogeneity, Including Shiga Toxin Encoding Phage stx2. PLoS ONE 7(11):E48228.
21. Moriel DG, et al. (2010) Identification of protective and broadly conserved vaccine antigens from the genome of extraintestinal pathogenic *Escherichia coli*. Proc Natl Acad Sci U S A 107(20):9072-9077.
22. Krause DO, et al. (2010) Complete genome sequence of adherent invasive *Escherichia coli* UM146 isolated from ileal Crohn's disease biopsy tissue. J Bacteriol 193(2):583.
23. Turner PC, et al. (2012) Optical mapping and sequencing of the *Escherichia coli* KO11 genome reveal extensive chromosomal rearrangements, and multiple tandem copies of the *Zymomonas mobilis pdc* and *adhB* genes. J Ind Microbiol Biotechnol 39(4):629-639.
24. Zdziarski J, et al. (2010) Host imprints on bacterial genomes--rapid, divergent evolution in individual patients. PLos Pathog 6(8):e1001078.
25. Touchon M, et al. (2009) Organised genome dynamics in the *Escherichia coli* species results in highly diverse adaptive paths. PLoS Genet 5(1):e1000344.
26. Blattner FR, et al. (1997) The complete genome sequence of *Escherichia coli* K-12. Science 277(5331):1453-1474 .
27. Iguchi A, et al. (2008) The complete genome sequence and comparative genome analysis of enteropathogenic *E. coli* O127:H6 strain E2348/69. J Bacteriol 191(1):347-354.
28. Welch RA, et al. (2002) Extensive mosaic structure revealed by the complete genome sequence of uropathogenic *Escherichia coli*. Proc Natl Acad Sci U S A 99(26):17020-17024.
29. Brzuszkiewicz E, et al. (2006) How to become a uropathogen: comparative genomic analysis of extraintestinal pathogenic *Escherichia coli* strains. Proc Natl Acad Sci U S A 103(34):12879-12884.
30. Chen SL, et al. (2006) Identification of genes subject to positive selection in uropathogenic strains of *Escherichia coli*: a comparative genomics approach. Proc Natl Acad Sci U S A 103(15):5977-5982.
31. Fiedoruk K, et al. (2014) First Complete Genome Sequence of *Escherichia albertii* Strain KF1, a New Potential Human Enteric Pathogen. Genome announc 2(1):e00004-14.

Table S3. Prophages and integrative elements of strains CB9786, NIAH_Bird_3 and EC06-170

Name	Position (start)	Position (end)	Length (bp)	Integration site	Length of *attL*/*R* (bp)	Type/ feature	Virulence-related genes and other notable genes
**CB9786**							
**Prophages**							
EACB_P01	267083	307492	40410	tRNA (*thrW*)	47	lambda-like	*bor*
EACB_P02	864592	899708	35117	*rcdA*	25	P2-like	
EACB_P03	961411	991516	30106	*pflA-pflB*	13	P2-like	
EACB_P04	1059619	1101689	42071	tRNA (*serT*)	65	lambda-like	*nleG*-family protein, hydrolase, *ompT*
EACB_P05	2033614	2081175	47562	*yegD-mdtA*	ND	lambda-like	*lom*, 3 *nleG*-family proteins, *nleA*/*EspI*-homolog
EACB_P06	2578454	2619827	41374	*yfhH-yfhL*	18	P2-like	
EACB_P07	4523112	4568340	45229	*yjjG-prfC*	14	lambda-like	*lom*
**Integrative elements**							
EACB_IE01	307540	320111	12572	tRNA (*thrW*)	20		
EACB_IE02	1244455	1258743	14289	*icd-minE*	ND		*nleG*-family protein, hydrolase, *ompT,* predicted adhesin-like autotransporter
EACB_IE03	1403363	1410580	7218	*sapC-sapB*	11		*emrE*, hydrolase
EACB_IE04	1960346	1964043	3698	tRNA (*asnV*)	15		
EACB_IE05	3039917	3062761	22845	tRNA (*pheV*)	ND		
EACB_IE06	3762095	3781365	19271	tRNA (*selC*)	ND		
EACB_IE07	4277029	4319065	42037	tRNA (*pheU*)	ND	LEE	T3SS machinery, *ibe*, *espG*, *espZ*, *espH*, *map*, *tir*, *espB*, *espF*, *espM*-homolog, *nleG*-family protein, *espK*-homolog, *espO*-homolog
EACB_IE08	4455929	4473494	17566	tRNA (*leuX*)	ND		*nleG*-family protein, autotransporter serine protease
**NIAH_Bird_3**							
**Prophages**							
EANB_P01	839729	874899	35171	*rcdA*	25	P2-like	
EANB_P02	1874934	1923648	48715	tRNA (*serU*)	32	lambda-like	tRNA(Gly [TCC], Thr [TGT], Met [CAT]), *espJ*-homolog[a], *tccP2*-homolog, *nleC*-homolog, *espX*-homolog, *nleG*-family protein
EANB_P03	3701451	3712143	10693	tRNA (*selC*)	23	P4-like	
EANB_P04	4004256	4036901	32646	*cpxP-fieF*	47	P2-like	
**Integrative elements**							
EANB_IE01	1161789	1180078	18290	*icd-minE*	ND		*nleG*-family protein, hydrolase, *ompT,* predicted adhesin-like autotransporter
EANB_IE02	1325385	1332602	7218	*sapC-sapB*	11		*emrE*, hydrolase
EANB_IE03	1932359	1944540	12182	tRNA (*asnT*)	20		
EANB_IE04	1949743	1953442	3700	tRNA (*asnU*)	15		
EANB_IE05	3712144	3732112	19969	tRNA (*asnU*)	ND		predicted adhesin
EANB_IE06	4288430	4328536	40107	tRNA (*pheU*)	ND	LEE	T3SS machinery, *ibe*, *espG*, *espZ*, *espH*, *map*, *tir*, *espB*, *espF*, *espM*-homolog, *nleG*-family protein, *espK*-homolog, *espO*-homolog
EANB_IE07	4467038	4478895	11858	tRNA (*leuX*)	ND		*nleB*, *nleE*, *nleG*-family protein, autotransporter serine protease
**EC06-170**							
**Prophages**							
EAEC_P01	878587	913183	34597	*rcdA*	20	P2-like	
EAEC_P02	1323565	1366341	42777	*ompW-trpA*	20	lambda-like	tRNA(Thr [TGT], Gly [TCC]), 3 *nleG*-family proteins, *espW*-homolog[a], *espM*-homolog
EAEC_P03	1971394	2020333	48940	tRNA (*serU*)	13	lambda-like	tRNA(Gly [TCC], Thr [TGT], Met [CAT]), *espJ*-homolog[a], *tccP2*-homolog, *nleC*-homolog, *espX*-homolog, *nleG*-family protein
EAEC_P04	2105356	2141946	36591	*yegD-mdtA*	34	lambda-like	*lom*, *nleG*-homolog, *nleA*/*EspI*-homolog
EAEC_P05	4231127	4278354	47228	*zur-dusA*	8	lambda-like	tRNA(Met [CAT], Thr [TGT], Gly [TCC]), *lom*, 4 *nleG*-family proteins, *espW*-homolog[a], *espM*-homolog
**Integrative elements**							
EAEC_IE01	334779	347190	12412	tRNA (*thrW*)	14		
EAEC_IE02	1201236	1215636	14401	*icd-minE*	ND		*nleG*-family protein, hydrolase, *ompT,* predicted adhesin-like autotransporter
EAEC_IE03	1410655	1417872	7218	*sapC-sapB*	11		*emrE*, hydrolase
EAEC_IE04	2177894	2185543	7650	*thiM-rcnR*	81		
EAEC_IE05	3802021	3820660	18640	tRNA (*selC*)	ND		predicted adhesin
EAEC_IE06	4360568	4401000	40433	tRNA (*pheU*)	ND	LEE	T3SS machinery, *ibe*, *espG*, *espZ*, *espH*, *map*, *tir*, *espB*, *espF*, *espM*-homolog, *nleG*-family protein, *espK*-homolog, *espO*-homolog
EAEC_IE07	4536404	4566214	22225	tRNA (*leuX*)	ND		*nleG*-family protein, autotransporter serine protease

ND: not detected

[a]: pseudogene

Table S4. IS elements of the three *E. albertii* strains completely sequenced in this study

IS element	Number of copies[a]		
	CB9786	NIAH_Bird_3	EC06-170
IS421	2	2	0
ISEc1	1	2 (1)	1
IS911	0	0	4
ISEal1[b]	1	0	0
ISL3-family IS element 1	1 (1)	0	0
ISL3-family IS element 2	1 (1)	2	0
ISAs1-family IS element 1	1 (1)	0	0
ISAs1-family IS element 2	1 (1)	1 (1)	0
ISAs1-family IS element 3	0	0	1 (1)
ISAs1-family IS element 4	0	0	1 (1)
ISAs1-family IS element 5	0	0	2 (1)
ISAs1-family IS element 6	0	0	1 (1)
ISAs1-family IS element 7	0	0	1
total	8 (4)	7 (2)	11 (4)

[a] Numbers of truncated or degraded copies are shown in parentheses.

[b] Newly identified IS elements.

Table S5A. *Escherichia albertii*-specific CDSs that are highly conserved in *E. albertii* but are absent or divergent in sequence in other *Escherichia* species/clades

Locus tag of CB9786	product	amino acid length	note[a]
EACBF0116	predicted protein	86	no homologues in *E. coli*
EACBF0348	predicted protein	188	no homologues in *E. coli*
EACBF0407	predicted protein	506	no homologues in *E. coli*
EACBF0500	predicted fimbrial-like adhesin protein	180	homolog (75% ID/99% LC) present in 29 *E. coli* strains
EACBF0504	predicted fimbrial-like adhesin protein	172	homolog (72-78% ID/99% LC) present in 30 *E. coli* strains
EACBF0506	predicted protein	163	no homologues in *E. coli*
EACBF0528	GNAT family acetyltransferase	234	no homologues in *E. coli*
EACBF0679	predicted protein	314	homolog (63-65% ID/100% LC) present in 13 *E. coli* strains
EACBF0741	predicted protein	118	homolog (66% ID/95% LC) present only in *E. coli* strain 042
EACBF1075	predicted protein	389	no homologues in *E. coli*
EACBF1099	predicted protein	103	homolog (65-67% ID/99% LC) present in 43 *E. coli* strains
EACBF1192	putative outer membrane protease	315	homolog (77% ID/100% LC) present in 29 *E. coli* strains
EACBF1240	predicted cytoplasmic protein	376	homolog (69-72% ID/97-99% LC) present in 18 *E. coli* strains
EACBF1245	predicted protein	345	no homologues in *E. coli*
EACBF1284	predicted ADP-heptose:LPS heptosyltransferase	352	homolog (65-70% ID/99% LC) present in 41 *E. coli* strains
EACBF1318	predicted protein	375	homolog (77-79% ID/99% LC) present in 44 *E. coli* strains
EACBF1328	predicted integrase	409	no homologues in *E. coli*
EACBF1370	T3SS secreted effetor NleG-like protein	140	no homologues in *E. coli*
EACBF1371	predicted protein	125	no homologues in *E. coli*
EACBF1389	predicted protein	125	homolog (74-78% ID/98% LC) present in 8 *E. coli* strains
EACBF1465	predicted protein	371	homolog (68% ID/100% LC) present in 19 *E. coli* strains
EACBF1496	predicted oxidoreductase, membrane subunit	285	homolog (78-80% ID/100% LC) present in 40 *E. coli* strains
EACBF1622	nicotinate-nucleotide--dimethylbenzimidazole phosphoribosyltransferase	354	homolog (57-58% ID/99% LC) present in 44 *E. coli* strains
EACBF1623	bifunctional cobinamide kinase/ cobinamide phosphate guanylyltransferase	183	homolog (56-57% ID/99% LC) present in 44 *E. coli* strains
EACBF1727	T3SS secreted effector protein NleF	144	homolog (55% ID/94% LC) present in 10 *E. coli* strains
EACBF1733	predicted protein	106	no homologues in *E. coli*
EACBF1757	predicted protein	228	no homologues in *E. coli*
EACBF1822	flagellar hook-length control protein	371	homolog (67-70% ID/100% LC) present in 44 *E. coli* strains
EACBF1922	predicted colanic acid biosynthesis protein, fragment	66	homolog (60-62% ID/94-99% LC) present in 42 *E. coli* strains
EACBF2009	cytolethal distending toxin A subunit	258	no homologues in *E. coli*
EACBF2010	cytolethal distending toxin B subunit	269	homolog (56-57% ID/99% LC) present in 2 *E. coli* strains (IHE30 and APEC O1)
EACBF2102	fumarate reductase, flaboprotein subunit	136	no homologues in *E. coli*
EACBF2104	predicted cytochrome c-type protein	194	homolog (50% ID/86% LC) present in 44 *E. coli* strains
EACBF2108	predicted autotransporter barrel domain-containing protein	862	homolog (75-76% ID/86-100% LC) present in 30 *E. coli* strains
EACBF2411	predicted acetyltransferase	193	no homologues in *E. coli*
EACBF2494	putative transcriptional regulator, AraC family	304	no homologues in *E. coli*
EACBF2495	Predicted acyl-CoA transferase	404	no homologues in *E. coli*
EACBF2498	predicted acyl-CoA dehydrogenase	386	homolog (67-68% ID/98% LC) present in 44 *E. coli* strains
EACBF2499	predicted electron transfer flavoprotein beta subunit	254	homolog (61-63% ID/100% LC) present in 44 *E. coli* strains

EACBF2501	predicted major facilitator superfamily protein	463	no homologues in *E. coli*
EACBF2539	putative alpha amylase	738	homolog (59-60% ID/91-94% LC) present in 20 *E. coli* strains
EACBF2935	predicted protein	82	no homologues in *E. coli*
EACBF2945	predicted protein	352	no homologues in *E. coli*
EACBF2946	predicted protein	136	no homologues in *E. coli*
EACBF3043	predicted protein	62	no homologues in *E. coli*
EACBF3044	predicted protein	70	no homologues in *E. coli*
EACBF3058	predicted protein	155	no homologues in *E. coli*
EACBF3272	predicted fimbrial assembly protein	179	homolog (76-77% ID/100% LC) present in 44 *E. coli* strains
EACBF3289	predicted transposase, central part	78	no homologues in *E. coli*
EACBF3454	alpha-amylase, N-ter part	56	homolog (64% ID/100% LC) present in 6 *E. coli* strains
EACBF3543	predicted protein	97	homolog (77-79% ID/100% LC) present in 44 *E. coli* strains
EACBF3766	predicted protein	351	homolog (69-80% ID/99% LC) present in 29 *E. coli* strains
EACBF4094	predicted protein	385	no homologues in *E. coli*
EACBF4169	predicted protein	273	homolog (61-63% ID/82-100% LC) present in 33 *E. coli* strains
EACBF4174	predicted protein	736	no homologues in *E. coli*

[a] ID: identity; LC: length coverage

Table S5B. *E. albertii* species-specific genomic regions (>100 bp)

Coordinates to the CB9786 genome sequence		
start	end	length (bp)
33174	33598	425
134204	134952	749
219227	219452	226
358511	358730	220
359983	360738	756
371946	372124	179
397524	397669	146
425047	426939	1893
548700	552284	3585
580508	581380	873
649204	649663	460
745367	746258	892
754247	754520	274
789786	791084	1299
807779	808489	711
853747	854503	757
1000002	1000107	106
1032319	1032485	167
1132866	1134337	1472
1171074	1171263	190
1219519	1219633	115

1250415	1251718	1304
1252519	1252684	166
1253575	1253847	273
1255894	1257381	1488
1258573	1258743	171
1263950	1264259	310
1265694	1266123	430
1266808	1266934	127
1270210	1270351	142
1285539	1286324	786
1289577	1289726	150
1302106	1305591	3486
1312208	1312368	161
1338241	1338448	208
1348645	1349567	923
1354965	1356009	1045
1357926	1358330	405
1368890	1369407	518
1403363	1404209	847
1405268	1405435	168
1406907	1407099	193
1408293	1408426	134
1410451	1410580	130
1438689	1438795	107
1438972	1439264	293
1448856	1449592	737
1480269	1480469	201
1491646	1491761	116
1506273	1506763	491
1510010	1510697	688
1527443	1527551	109
1594299	1594408	110
1627985	1628116	132
1634093	1634198	106
1734330	1735874	1545
1739959	1740893	935
1776689	1777643	955
1806910	1807170	261
1841811	1842274	464
1849169	1849377	209
1865514	1866519	1006
1885863	1886130	268

1896612	1896855	244
1943588	1943775	188
1950123	1950397	275
1952900	1953626	727
1955186	1955353	168
1965114	1965227	114
2021671	2022247	577
2185316	2185426	111
2208930	2212094	3165
2304568	2304703	136
2343381	2343486	106
2383510	2383646	137
2387500	2387848	349
2388157	2388261	105
2418546	2418919	374
2501123	2501397	275
2550928	2551153	226
2639910	2643977	4068
2689688	2689797	110
2689976	2692789	2814
2702191	2702463	273
2821561	2821675	115
2880486	2880585	100
2910128	2910894	767
2911801	2911900	100
2958494	2958652	159
2987936	2988984	1049
2999773	3000085	313
3114254	3114499	246
3115636	3115795	160
3125519	3126093	575
3136352	3137942	1591
3157479	3157856	378
3244537	3244961	425
3258662	3260172	1511
3293331	3293484	154
3469001	3469100	100
3600961	3601152	192
3609237	3609428	192
3615465	3615629	165
3668798	3668936	139
3670782	3670919	138

3690531	3691336	806
3698112	3698278	167
3738872	3739104	233
3761692	3761892	201
3762279	3762460	182
3764821	3766751	1931
3792490	3792646	157
4065949	4066596	648
4396916	4397294	379
4402572	4404078	1507
4490309	4491425	1117
4494827	4497617	2791
4516576	4516832	257
	total length	71280

Table S6. *E. coli* K-12 genes that are absent in all of the three *E. albertii* strains completely sequenced in this study

locus tag in the *E. coli* K-12 MG1655 genome	direction	gene	product	Prevalence in the 44 fully sequenced *E. coli* strains
b0070	+	*setA*	broad specificity sugar efflux system	40.9%
b0135	-	*yadC*	predicted fimbrial-like adhesin protein	6.8%
b0136	-	*yadK*	predicted fimbrial-like adhesin protein	6.8%
b0137	-	*yadL*	predicted fimbrial-like adhesin protein	40.9%
b0138	-	*yadM*	predicted fimbrial-like adhesin protein	38.6%
b0140	-	*ecpD*	predicted periplasmic pilin chaperone	45.5%
b0141	-	*yadN*	predicted fimbrial-like adhesin protein	43.2%
b0193	-	*yaeF*	predicted lipoprotein	84.1%
b0210	+	*yafE*	predicted S-adenosyl-L-methionine-dependent methyltransferase	100.0%
b0225	-	*yafQ*	predicted toxin of the YafQ-DinJ toxin-antitoxin system	50.0%
b0226	-	*dinJ*	predicted antitoxin of YafQ-DinJ toxin-antitoxin system	59.1%
b0245	-	*ykfI*	CP4-6 prophage; toxin of the YkfI-YafW toxin-antitoxin system	4.5%
b0246	-	*yafW*	CP4-6 prophage; antitoxin of the YkfI-YafW toxin-antitoxin system	4.5%
b4504	-	*ykfH*	predicted protein	2.3%
b0247	-	*ykfG*	CP4-6 prophage; predicted DNA repair protein	4.5%
b0248	-	*yafX*	CP4-6 prophage; predicted protein	4.5%
b0249	-	*ykfF*	CP4-6 prophage; predicted protein	2.3%
b0250	-	*ykfB*	CP4-6 prophage; predicted protein	4.5%
b0253	-	*ykfA*	CP4-6 prophage; predicted GTP-binding protein	4.5%
b0254	-	*perR*	CP4-6 prophage; predicted DNA-binding transcriptional regulator	2.3%
b0260	+	*mmuP*	CP4-6 prophage; predicted S-methylmethionine transporter	4.5%
b0261	+	*mmuM*	CP4-6 prophage; S-methylmethionine:homocysteine methyltransferase	4.5%
b0262	-	*afuC*	CP4-6 prophage; predicted ferric transporter subunit	22.7%
b0267	-	*yagA*	CP4-6 prophage; predicted DNA-binding transcriptional regulator	2.3%
b0268	+	*yagE*	CP4-6 prophage; predicted lyase/synthase	2.3%
b0269	+	*yagF*	CP4-6 prophage; predicted dehydratase	2.3%
b0270	+	*yagG*	CP4-6 prophage; predicted sugar transporter	2.3%
b0271	+	*yagH*	CP4-6 prophage; predicted xylosidase/arabinosidase	2.3%
b0272	-	*yagI*	CP4-6 prophage; predicted DNA-binding transcriptional regulator	2.3%
b0277	-	*yagK*	CP4-6 prophage; conserved protein	2.3%
b0278	-	*yagL*	CP4-6 prophage; DNA-binding protein	2.3%
b0279	-	*yagM*	CP4-6 prophage; predicted protein	2.3%
b0280	-	*yagN*	CP4-6 prophage; predicted protein	2.3%
b0281	-	*intF*	CP4-6 prophage; predicted phage integrase	2.3%
b0283	-	*yagQ*	conserved protein	52.3%
b0284	-	*yagR*	predicted oxidoreductase with molybdenum-binding domain	54.5%
b0285	-	*yagS*	predicted oxidoreductase with FAD-binding domain	54.5%
b0286	-	*yagT*	predicted xanthine dehydrogenase, 2Fe-2S subunit	52.3%
b0289	-	*yagV*	conserved protein	90.9%
b0290	-	*yagW*	predicted receptor	90.9%
b0291	-	*yagX*	predicted aromatic compound dioxygenase	93.2%
b0292	-	*matC*	predicted protein	93.2%
b0293	-	*matB*	conserved protein	90.9%
b0294	-	*matA*	predicted regulator	95.5%
b0295	+	*ykgL*	predicted protein	22.7%
b0310	-	*ykgH*	predicted inner membrane protein	81.8%
b0311	-	*betA*	choline dehydrogenase, a flavoprotein	97.7%
b0312	-	*betB*	betaine aldehyde dehydrogenase, NAD-dependent	97.7%
b0313	-	*betI*	DNA-binding transcriptional repressor	97.7%
b0314	+	*betT*	choline transporter of high affinity	95.5%
b0325	+	*yahK*	predicted oxidoreductase, Zn-dependent and NAD(P)-binding	95.5%
b0326	+	*yahL*	predicted protein	68.2%
b0327	+	*yahM*	predicted protein	34.1%
b0336	+	*codB*	cytosine transporter	100.0%
b0337	+	*codA*	cytosine deaminase	100.0%
b0338	-	*cynR*	DNA-binding transcriptional dual regulator	68.2%
b0339	+	*cynT*	carbonic anhydrase	68.2%
b0340	+	*cynS*	cyanate aminohydrolase	68.2%
b0341	+	*cynX*	predicted cyanate transporter	68.2%
b0342	-	*lacA*	thiogalactoside acetyltransferase	84.1%
b0343	-	*lacY*	lactose/galactose transporter	100.0%
b0345	-	*lacI*	DNA-binding transcriptional repressor	97.7%
b0346	-	*mhpR*	DNA-binding transcriptional activator, 3HPP-binding	75.0%
b0347	+	*mhpA*	3-(3-hydroxyphenyl)propionate hydroxylase	75.0%
b0348	+	*mhpB*	2,3-dihydroxyphenylpropionate 1,2-dioxygenase	75.0%
b0349	+	*mhpC*	2-hydroxy-6-ketonona-2,4-dienedioic acid hydrolase	75.0%
b0350	+	*mhpD*	2-keto-4-pentenoate hydratase	75.0%
b0351	+	*mhpF*	acetaldehyde-CoA dehydrogenase II, NAD-binding	75.0%
b0352	+	*mhpE*	4-hyroxy-2-oxovalerate/4-hydroxy-2-oxopentanoic acid aldolase, class I	75.0%
b0353	+	*mhpT*	predicted 3-hydroxyphenylpropionic transporter	75.0%
b0358	-	*yaiO*	predicted protein	79.5%

b0363	-	*yaiP*	predicted glucosyltransferase	79.5%
b0364	-	*yaiS*	conserved protein	72.7%
b0389	+	*yaiA*	predicted protein	95.5%
b0396	-	*araJ*	predicted transporter	97.7%
b0498	+	*ybbC*	predicted protein	27.3%
b0504	-	*allS*	DNA-binding transcriptional activator of the allD operon	97.7%
b0505	+	*allA*	ureidoglycolate hydrolase	97.7%
b0506	+	*allR*	DNA-binding transcriptional repressor	81.8%
b0507	+	*gcl*	glyoxylate carboligase	97.7%
b0508	+	*hyi*	hydroxypyruvate isomerase	95.5%
b0509	+	*glxR*	tartronate semialdehyde reductase, NADH-dependent	97.7%
b0511	+	*ybbW*	predicted allantoin transporter	95.5%
b0512	+	*allB*	allantoinase	88.6%
b0513	+	*ybbY*	predicted uracil/xanthine transporter	95.5%
b0514	+	*glxK*	glycerate kinase II	100.0%
b0515	-	*ylbA*	conserved protein	100.0%
b0516	-	*allC*	allantoate amidohydrolase	97.7%
b0517	-	*allD*	ureidoglycolate dehydrogenase	100.0%
b0518	+	*fdrA*	predicted acyl-CoA synthetase with NAD(P)-binding Rossmann-fold domain	97.7%
b0520	+	*ylbF*	conserved protein	100.0%
b0521	+	*ybcF*	predicted carbamate kinase	100.0%
b0544	+	*ybcK*	DLP12 prophage; predicted recombinase	27.3%
b4588	+	*ylcH*	hypothetical protein, DLP12 prophage	4.5%
b0547	+	*ybcN*	DLP12 prophage; predicted protein	54.5%
b0548	+	*ninE*	DLP12 prophage; conserved protein	40.9%
b0549	+	*ybcO*	DLP12 prophage; predicted protein	50.0%
b0550	+	*rusA*	DLP12 prophage; endonuclease RUS	70.5%
b4509	+	*ylcG*	DLP12 prophage; predicted protein	31.8%
b0551	+	*ybcQ*	DLP12 prophage; predicted antitermination protein	59.1%
b0558	-	*ybcV*	DLP12 prophage; predicted protein	22.7%
b0559	+	*ybcW*	DLP12 prophage; predicted protein	29.5%
b0564	+	*appY*	DLP12 prophage; DNA-binding transcriptional activator	4.5%
b0581	-	*ybdK*	gamma-glutamyl:cysteine ligase	100.0%
b0608	+	*ybdR*	predicted oxidoreductase, Zn-dependent and NAD(P)-binding	54.5%
b0689	+	*ybfP*	predicted protein	25.0%
b0730	-	*mngR*	DNA-binding transcriptional dual regulator, fatty-acyl-binding	47.7%
b0731	+	*mngA*	fused 2-O-a-mannosyl-D-glycerate specific PTS enzymes: IIA component/IIB component/IIC com	43.2%
b0732	+	*mngB*	alpha-mannosidase	43.2%
b0753	-	*ybgS*	conserved protein	100.0%
b0788	-	*ybhN*	conserved inner membrane protein	100.0%
b0789	-	*ybhO*	cardiolipin synthase 2	100.0%
b0790	-	*ybhP*	predicted DNase	100.0%
b0804	-	*ybiX*	conserved protein	100.0%
b0805	-	*fiu*	predicted iron outer membrane transporter	97.7%
b0808	-	*ybiO*	predicted mechanosensitive channel	100.0%
b0816	+	*yliL*	predicted protein	4.5%
b0821	-	*ybiU*	predicted protein	59.1%
b0837	+	*yliI*	predicted dehydrogenase	97.7%
b0900	-	*ycaN*	predicted DNA-binding transcriptional regulator	43.2%
b0901	+	*ycaK*	conserved protein	43.2%
b0906	+	*ycaP*	conserved inner membrane protein	100.0%
b0909	+	*ycaL*	predicted peptidase with chaperone function	100.0%
b0933	-	*ssuB*	alkanesulfonate transporter subunit	100.0%
b0934	-	*ssuC*	alkanesulfonate transporter subunit	100.0%
b0935	-	*ssuD*	alkanesulfonate monooxygenase, FMNH(2)-dependent	100.0%
b0936	-	*ssuA*	alkanesulfonate transporter subunit	97.7%
b0937	-	*ssuE*	NAD(P)H-dependent FMN reductase	100.0%
b0938	+	*ycbQ*	predicted fimbrial-like adhesin protein	34.1%
b0939	+	*ycbR*	predicted periplasmic pilin chaperone	63.6%
b0940	+	*ycbS*	predicted outer membrane usher protein	47.7%
b0941	+	*ycbT*	predicted fimbrial-like adhesin protein	54.5%
b0942	+	*ycbU*	predicted fimbrial-like adhesin protein	61.4%
b0943	+	*ycbV*	predicted fimbrial-like adhesin protein	63.6%
b0944	+	*ycbF*	predicted periplasmic pilini chaperone	52.3%
b4592	+	*yccB*	hypothetical protein	31.8%
b0987	-	*gfcA*	predicted protein	52.3%
b0991	+	*ymcE*	cold shock gene	61.4%
b1001	+	*yccE*	predicted protein	11.4%
b1006	-	*rutG*	predicted transporter	88.6%
b1007	-	*rutF*	predicted oxidoreductase, flavin:NADH component	100.0%
b1008	-	*rutE*	predicted oxidoreductase	100.0%
b1009	-	*rutD*	predicted hydrolase	100.0%
b1010	-	*rutC*	conserved protein	95.5%
b1011	-	*rutB*	predicted enzyme	97.7%

b1012	-	*rutA*	predicted monooxygenase	100.0%
b1021	-	*pgaD*	predicted inner membrane protein	79.5%
b1022	-	*pgaC*	predicted glycosyl transferase	79.5%
b1023	-	*pgaB*	predicted enzyme associated with biofilm formation	77.3%
b1024	-	*pgaA*	predicted outer membrane protein	72.7%
b1029	+	*ycdU*	predicted inner membrane protein	47.7%
b1056	-	*yceI*	predicted protein	100.0%
b1057	-	*yceJ*	predicted cytochrome b561	100.0%
b1064	-	*grxB*	glutaredoxin 2 (Grx2)	100.0%
b1102	-	*fhuE*	ferric-rhodotorulic acid outer membrane transporter	97.7%
b1115	-	*ycfT*	predicted inner membrane protein	100.0%
b1137	-	*ymfD*	e14 prophage; predicted SAM-dependent methyltransferase	2.3%
b1138	-	*ymfE*	e14 prophage; predicted inner membrane protein	2.3%
b1139	+	*lit*	e14 prophage; cell death peptidase, inhibitor of T4 late gene expression	2.3%
b1141	-	*xisE*	e14 prophage; predicted excisionase	2.3%
b1143	+	*ymfI*	e14 prophage; predicted protein	2.3%
b1144	-	*ymfJ*	e14 prophage; predicted protein	2.3%
b1159	+	*mcrA*	e14 prophage; 5-methylcytosine-specific restriction endonuclease B	2.3%
b1160	-	*elbA*	predicted protein	43.2%
b1162	-	*ycgE*	predicted DNA-binding transcriptional regulator	86.4%
b1163	-	*ycgF*	predicted FAD-binding phosphodiesterase	77.3%
b1164	+	*ycgZ*	predicted protein	68.2%
b1165	+	*ymgA*	predicted protein	63.6%
b1166	+	*ymgB*	predicted protein	79.5%
b1167	+	*ymgC*	predicted protein	72.7%
b1168	+	*ycgG*	conserved inner membrane protein	52.3%
b4593	-	*ymgI*	hypothetical protein	25.0%
b4594	+	*ymgJ*	hypothetical protein	11.4%
b1177	+	*ycgJ*	predicted protein	100.0%
b1182	-	*hlyE*	hemolysin E	38.6%
b1183	+	*umuD*	DNA polymerase V, subunit D	100.0%
b1184	+	*umuC*	DNA polymerase V, subunit C	97.7%
b1202	-	*ycgV*	predicted adhesin	45.5%
b4423	-	*ldrC*	toxic polypeptide, small	81.8%
b1217	+	*chaB*	predicted cation regulator	97.7%
b1220	+	*ychO*	predicted invasin	100.0%
b1229	-	*tpr*	predicted protamine-like protein	6.8%
b4595	+	*yciY*	hypothetical protein	56.8%
b1257	-	*yciE*	conserved protein	88.6%
b1258	-	*yciF*	conserved protein	90.9%
b1268	+	*yciQ*	predicted inner membrane protein	54.5%
b4522	+	*ymiA*	hypothetical protein	86.4%
b4523	+	*yciX*	hypothetical protein	84.1%
b1309	+	*ycjM*	predicted glucosyltransferase	77.3%
b1310	+	*ycjN*	predicted sugar transporter subunit: periplasmic-binding component of ABC superfamily	84.1%
b1311	+	*ycjO*	predicted sugar transporter subunit: membrane component of ABC superfamily	93.2%
b1312	+	*ycjP*	predicted sugar transporter subunit: membrane component of ABC superfamily	95.5%
b1313	+	*ycjQ*	predicted oxidoreductase, Zn-dependent and NAD(P)-binding	93.2%
b1314	+	*ycjR*	predicted enzyme	95.5%
b1315	+	*ycjS*	predicted oxidoreductase, NADH-binding	93.2%
b1316	+	*ycjT*	predicted hydrolase	90.9%
b1317	+	*ycjU*	predicted beta-phosphoglucomutase	93.2%
b1319	+	*ompG*	outer membrane porin	93.2%
b1320	-	*ycjW*	predicted DNA-binding transcriptional regulator	95.5%
b1336	-	*abgT*	predicted cryptic aminobenzoyl-glutamate transporter	59.1%
b1337	-	*abgB*	predicted peptidase, aminobenzoyl-glutamate utilization protein	65.9%
b1338	-	*abgA*	predicted peptidase, aminobenzoyl-glutamate utilization protein	68.2%
b1339	+	*abgR*	predicted DNA-binding transcriptional regulator	68.2%
b1345	-	*intR*	Rac prophage; integrase	25.0%
b1346	-	*ydaQ*	Rac prophage; conserved protein	22.7%
b1347	-	*ydaC*	Rac prophage; predicted protein	25.0%
b1348	-	*lar*	Rac prophage; restriction alleviation protein	15.9%
b1349	-	*recT*	Rac prophage; recombination and repair protein	20.5%
b1350	-	*recE*	Rac prophage; exonuclease VIII, 5' -> 3' specific dsDNA exonuclease	15.9%
b1351	-	*racC*	Rac prophage; predicted protein	20.5%
b4526	-	*ydaE*	Rac prophage; conserved protein	6.8%
b1353	+	*sieB*	Rac prophage; phage superinfection exclusion protein	13.6%
b1355	-	*ydaG*	Rac prophage; predicted protein	13.6%
b1356	-	*racR*	Rac prophage; predicted DNA-binding transcriptional regulator	18.2%
b1357	+	*ydaS*	Rac prophage; predicted DNA-binding transcriptional regulator	4.5%
b1358	+	*ydaT*	Rac prophage; predicted protein	20.5%
b1365	+	*ynaK*	Rac prophage; conserved protein	2.3%
b1384	-	*feaR*	DNA-binding transcriptional dual regulator	40.9%
b1385	+	*feaB*	phenylacetaldehyde dehydrogenase	38.6%

b1386	-	*tynA*	tyramine oxidase, copper-requiring	40.9%
b1387	-	*maoC*	fused aldehyde dehydrogenase/enoyl-CoA hydratase	40.9%
b1388	+	*paaA*	predicted multicomponent oxygenase/reductase subunit for phenylacetic acid degradation	40.9%
b1389	+	*paaB*	predicted multicomponent oxygenase/reductase subunit for phenylacetic acid degradation	40.9%
b1390	+	*paaC*	predicted multicomponent oxygenase/reductase subunit for phenylacetic acid degradation	40.9%
b1391	+	*paaD*	predicted multicomponent oxygenase/reductase subunit for phenylacetic acid degradation	40.9%
b1392	+	*paaE*	predicted multicomponent oxygenase/reductase subunit for phenylacetic acid degradation	40.9%
b1393	+	*paaF*	enoyl-CoA hydratase-isomerase	40.9%
b1394	+	*paaG*	acyl-CoA hydratase	40.9%
b1395	+	*paaH*	3-hydroxybutyryl-CoA dehydrogenase	40.9%
b1396	+	*paaI*	predicted thioesterase	40.9%
b1397	+	*paaJ*	predicted beta-ketoadipyl CoA thiolase	40.9%
b1398	+	*paaK*	phenylacetyl-CoA ligase	40.9%
b1399	+	*paaX*	DNA-binding transcriptional repressor of phenylacetic acid degradation, aryl-CoA responsive	40.9%
b1400	+	*paaY*	predicted hexapeptide repeat acetyltransferase	43.2%
b1408	+	*ynbA*	predicted inner membrane protein	11.4%
b1409	+	*ynbB*	predicted CDP-diglyceride synthase	84.1%
b1410	+	*ynbC*	predicted hydrolase	88.6%
b1411	+	*ynbD*	predicted phosphatase, inner membrane protein	88.6%
b1415	+	*aldA*	aldehyde dehydrogenase A, NAD-linked	95.5%
b4428	-	*hokB*	toxic polypeptide, small	29.5%
b1420	-	*mokB*	regulatory peptide	38.6%
b1421	+	*trg*	methyl-accepting chemotaxis protein III, ribose and galactose sensor receptor	63.6%
b1432	+	*ydcM*	predicted transposase	75.0%
b1433	-	*ydcO*	predicted benzoate transporter	90.9%
b1434	+	*ydcN*	predicted DNA-binding transcriptional regulator	97.7%
b4532	+	*yncN*	predicted protein	18.2%
b1438	+	*ydcQ*	predicted DNA-binding transcriptional regulator	63.6%
b1450	+	*yncC*	predicted DNA-binding transcriptional regulator	65.9%
b1454	+	*yncG*	predicted enzyme	72.7%
b1455	+	*yncH*	predicted protein	40.9%
b1457	+	*ydcD*	predicted protein	27.3%
b1470	-	*yddJ*	predicted protein	6.8%
b1480	-	*sra*	30S ribosomal subunit protein S22	81.8%
b1481	-	*bdm*	biofilm-dependent modulation protein	100.0%
b1483	-	*ddpF*	D-ala-D-ala transporter subunit	65.9%
b1484	-	*ddpD*	D-ala-D-ala transporter subunit	38.6%
b1485	-	*ddpC*	D-ala-D-ala transporter subunit	68.2%
b1486	-	*ddpB*	D-ala-D-ala transporter subunit	68.2%
b1487	-	*ddpA*	D-ala-D-a la transporter subunit	63.6%
b1488	-	*ddpX*	D-ala-D-ala dipeptidase, Zn-dependent	65.9%
b1494	-	*pqqL*	predicted peptidase	88.6%
b1495	-	*yddB*	predicted porin protein	88.6%
b1496	-	*yddA*	fused predicted multidrug transporter subunits of ABC superfamily: membrane component/ATP-bi	88.6%
b1499	-	*ydeO*	predicted DNA-binding transcriptional acfivator	95.5%
b1501	-	*ydeP*	predicted oxidoreductase	100.0%
b1502	-	*ydeQ*	predicted fimbrial-like adhesin protein	93.2%
b1503	-	*ydeR*	predicted fimbrial-like adhesin protein	84.1%
b1504	-	*ydeS*	predicted fimbrial-like adhesin protein	86.4%
b1507	-	*hipA*	regulator with hipB	81.8%
b1508	-	*hipB*	DNA-binding transcriptional regulator	81.8%
b1511	-	*lsrK*	autoinducer-2 (AI-2) kinase	61.4%
b1512	-	*lsrR*	lsr operon transcriptional repressor	65.9%
b1513	+	*lsrA*	fused AI2 transporter subunits of ABC superfamily: ATP-binding components	63.6%
b1514	+	*lsrC*	AI2 transporter	63.6%
b1515	+	*lsrD*	AI2 transporter	63.6%
b1516	+	*lsrB*	AI2 transporter	65.9%
b1517	+	*lsrF*	putative autoinducer-2 (AI-2) aldolase	65.9%
b1518	+	*lsrG*	autoinducer-2 (AI-2) modifying protein LsrG	61.4%
b4599	+	*yneM*	hypothetical protein	27.3%
b1536	-	*ydeI*	conserved protein	65.9%
b1537	+	*ydeJ*	conserved protein	56.8%
b1547	-	*ydfN*	Qin prophage; predicted side tail fibre assembly protein	2.3%
b1551	-	*ynfN*	Qin prophage; predicted protein	13.6%
b1553	-	*ydfP*	Qin prophage; conserved protein	31.8%
b1563	-	*relE*	Qin prophage; toxin of the RelE-RelB toxin-antitoxin system	15.9%
b1564	-	*relB*	Qin prophage; bifunctional antitoxin of the RelE-RelB toxin-antitoxin system/ transcriptional repre	20.5%
b1565	+	*ydfV*	Qin prophage; predicted protein	6.8%
b1566	+	*flxA*	Qin prophage; predicted protein	18.2%
b1572	+	*ydfB*	Qin prophage; predicted protein	52.3%
b1615	-	*uidC*	predicted outer membrane porin protein	90.9%
b1617	-	*uidA*	beta-D-glucuronidase	93.2%
b1618	-	*uidR*	DNA-binding transcriptional repressor	100.0%
b1646	-	*sodC*	superoxide dismutase, Cu, Zn	100.0%

b1657	-	*ydhP*	predicted transporter	100.0%
b4602	-	*ynhF*	hypothetical protein	54.5%
b1668	+	*ydhS*	conserved protein with FAD/NAD(P)-binding domain	93.2%
b1705	+	*ydiE*	conserved protein	95.5%
b1715	-	*pheM*	phenylalanyl-tRNA synthetase operon leader peptide	45.5%
b1730	-	*ydjO*	predicted protein	31.8%
b1732	+	*katE*	hydroperoxidase HPII(III) (catalase)	100.0%
b1741	+	*cho*	endonuclease of nucleotide excision repair	100.0%
b1762	-	*ynjI*	predicted inner membrane protein	50.0%
b1781	-	*yeaE*	predicted oxidoreductase	100.0%
b1790	-	*yeaM*	predicted DNA-binding transcriptional regulator	97.7%
b1791	+	*yeaN*	predicted transporter	100.0%
b1846	-	*yebE*	conserved protein	97.7%
b1877	+	*yecT*	predicted protein	54.5%
b1881	-	*cheZ*	chemotaxis regulator, protein phosphatase for CheY	100.0%
b1882	-	*cheY*	chemotaxis regulator transmitting signal to flagellar motor component	100.0%
b1883	-	*cheB*	fused chemotaxis regulator: protein-glutamate methylesterase in two-component regulatory system	100.0%
b1884	-	*cheR*	chemotaxis regulator, protein-glutamate methyltransferase	100.0%
b1885	-	*tap*	methyl-accepting protein IV	61.4%
b1886	-	*tar*	methyl-accepting chemotaxis protein II	100.0%
b1887	-	*cheW*	purine-binding chemotaxis protein	100.0%
b1923	-	*fliC*	flagellar filament structural protein (flagellin)	2.3%
b1931	+	*yedK*	predicted protein	77.3%
b1932	+	*yedL*	predicted acyltransferase	25.0%
b1943	+	*fliK*	flagellar hook-length control protein	100.0%
b1951	+	*rcsA*	DNA-binding transcriptional activator, co-regulator with RcsB	100.0%
b1974	+	*yodB*	predicted cytochrome	47.7%
b1978	+	*yeeJ*	adhesin	11.4%
b1983	+	*yeeN*	conserved protein	100.0%
b1991	-	*cobT*	nicotinate-nucleotide dimethylbenzimidazole-P phophoribosyl transferase	100.0%
b1993	-	*cobU*	bifunctional cobinamide kinase/ cobinamide phosphate guanylyltransferase	97.7%
b2000	+	*flu*	CP4-44 prophage; antigen 43 (Ag43) phase-variable biofilm formation autotransporter	9.1%
b2001	+	*yeeR*	CP4-44 prophage; predicted membrane protein	2.3%
b2002	+	*yeeS*	CP4-44 prophage; predicted DNA repair protein	81.8%
b2003	+	*yeeT*	CP4-44 prophage; predicted protein	77.3%
b2005	+	*yeeV*	CP4-44 prophage; toxin of the YeeV-YeeU toxin-antitoxin system	31.8%
b2012	-	*yeeD*	conserved protein	65.9%
b2013	-	*yeeE*	predicted inner membrane protein	70.5%
b4539	-	*yoeB*	toxin of the YoeB-YefM toxin-antitoxin system	72.7%
b2017	-	*yefM*	antitoxin of the YoeB-YefM toxin-antitoxin system	75.0%
b2032	-	*wbbK*	lipopolysaccharide biosynthesis protein	4.5%
b2033	-	*wbbJ*	predicted acyl transferase	4.5%
b2034	-	*wbbI*	conserved protein	4.5%
b2035	-	*rfc*	O-antigen polymerase	4.5%
b2036	-	*glf*	UDP-galactopyranose mutase, FAD/NAD(P)-binding	4.5%
b2037	-	*rfbX*	predicted polisoprenol-linked O-antigen transporter	4.5%
b2043	-	*wcaM*	predicted colanic acid biosynthesis protein	95.5%
b2045	-	*wcaK*	predicted pyruvyl transferase	95.5%
b2046	-	*wzxC*	colanic acid exporter	93.2%
b2047	-	*wcaJ*	predicted UDP-glucose lipid carrier transferase	95.5%
b2048	-	*cpsG*	phosphomannomutase	95.5%
b2049	-	*cpsB*	mannose-1-phosphate guanyltransferase	95.5%
b2050	-	*wcaI*	predicted glycosyl transferase	93.2%
b2052	-	*fcl*	bifunctional GDP-fucose synthetase: GDP-4-dehydro-6-deoxy-D-mannose epimerase/ GDP-4-deh	95.5%
b2053	-	*gmd*	GDP-D-mannose dehydratase, NAD(P)-binding	95.5%
b2055	-	*wcaE*	predicted glycosyl transferase	95.5%
b2056	-	*wcaD*	predicted colanic acid polymerase	97.7%
b2057	-	*wcaC*	predicted glycosyl transferase	95.5%
b2058	-	*wcaB*	predicted acyl transferase	95.5%
b2059	-	*wcaA*	predicted glycosyl transferase	95.5%
b2060	-	*wzc*	protein-tyrosine kinase	95.5%
b2070	-	*yegI*	conserved protein	97.7%
b2071	+	*yegJ*	predicted protein	22.7%
b2072	-	*yegK*	predicted protein	100.0%
b2073	-	*yegL*	conserved protein	100.0%
b2085	-	*yegR*	predicted protein	97.7%
b2091	-	*gatD*	galactitol-1-phosphate dehydrogenase, Zn-dependent and NAD(P)-binding	90.9%
b2092	-	*gatC*	galactitol-specific enzyme IIC component of PTS	90.9%
b2093	-	*gatB*	galactitol-specific enzyme IIB component of PTS	90.9%
b2094	-	*gatA*	galactitol-specific enzyme IIA component of PTS	93.2%
b2095	-	*gatZ*	D-tagatose 1,6-bisphosphate aldolase 2, subunit	84.1%
b2096	-	*gatY*	D-tagatose 1,6-bisphosphate aldolase 2, catalytic subunit	90.9%
b4542	+	*yohO*	predicted protein	45.5%
b2137	-	*yohF*	predicted oxidoreductase with NAD(P)-binding Rossmann-fold domain	97.7%

b2154	+	*yeiG*	predicted esterase	100.0%
b2162	-	*rihB*	ribonucleoside hydrolase 2	100.0%
b2163	+	*yeiL*	DNA-binding transcriptional activator of stationary phase nitrogen survival	100.0%
b4604	+	*yojO*	hypothetical protein	59.1%
b2237	-	*inaA*	conserved protein	100.0%
b2269	+	*elaD*	predicted enzyme	27.3%
b2273	-	*yfbN*	predicted protein	9.1%
b2274	+	*yfbO*	predicted protein	9.1%
b2275	+	*yfbP*	predicted protein	9.1%
b2301	-	*yfcF*	predicted enzyme	97.7%
b2332	-	*yfcO*	predicted protein	6.8%
b2334	-	*yfcQ*	predicted fimbrial-like adhesin protein	6.8%
b2335	-	*yfcR*	predicted fimbrial-like adhesin protein	6.8%
b2339	-	*yfcV*	predicted fimbrial-like adhesin protein	40.9%
b2345	+	*yfdF*	predicted protein	9.1%
b2347	+	*yfdC*	predicted inner membrane protein	100.0%
b2349	+	*intS*	CPS-53 (KpLE1) prophage; predicted prophage CPS-53 integrase	54.5%
b2350	+	*yfdG*	CPS-53 (KpLE1) prophage; bactoprenol-linked glucose translocase (flippase)	6.8%
b2351	+	*yfdH*	CPS-53 (KpLE1) prophage; bactoprenol glucosyl transferase	2.3%
b2352	+	*yfdI*	CPS-53 (KpLE1) prophage; predicted inner membrane protein	2.3%
b4501	+	*torI*	response regulator inhibitor for tor operon	31.8%
b2368	-	*emrK*	EmrKY-TolC multidrug resistance efflux pump, membrane fusion protein component	95.5%
b2369	+	*evgA*	DNA-binding response regulator in two-component regulatory system with EvgS	97.7%
b2370	+	*evgS*	hybrid sensory histidine kinase in two-component regulatory system with EvgA	95.5%
b2371	-	*yfdE*	predicted CoA-transferase, NAD(P)-binding	93.2%
b2372	-	*yfdV*	predicted transporter	97.7%
b2373	-	*oxc*	predicted oxalyl-CoA decarboxylase	97.7%
b2374	-	*frc*	formyl-CoA transferase, NAD(P)-binding	100.0%
b2375	-	*yfdX*	predicted protein	100.0%
b2376	+	*ypdI*	predicted lipoprotein involved in colanic acid biosynthesis	63.6%
b2408	+	*yfeN*	conserved outer membrane protein	61.4%
b2409	-	*yfeR*	predicted DNA-binding transcriptional regulator	86.4%
b2427	-	*yfeT*	predicted DNA-binding transcriptional regulator	68.2%
b2442	+	*intZ*	CPZ-55 prophage; predicted integrase	4.5%
b2443	+	*yffL*	CPZ-55 prophage; predicted protein	2.3%
b2444	+	*yffM*	CPZ-55 prophage; predicted protein	2.3%
b2445	+	*yffN*	CPZ-55 prophage; predicted protein	2.3%
b2446	+	*yffO*	CPZ-55 prophage; predicted protein	2.3%
b2447	+	*yffP*	CPZ-55 prophage; predicted protein	2.3%
b2448	+	*yffQ*	CPZ-55 prophage; predicted protein	2.3%
b2449	+	*yffR*	CPZ-55 prophage; predicted protein	2.3%
b2450	+	*yffS*	CPZ-55 prophage; predicted protein	2.3%
b2466	-	*ypfG*	predicted protein	100.0%
b4606	-	*ypfM*	hypothetical protein	2.3%
b2481	+	*hyfA*	hydrogenase 4, 4Fe-4S subunit	68.2%
b2482	+	*hyfB*	hydrogenase 4, membrane subunit	59.1%
b2483	+	*hyfC*	hydrogenase 4, membrane subunit	63.6%
b2484	+	*hyfD*	hydrogenase 4, membrane subunit	54.5%
b2485	+	*hyfE*	hydrogenase 4, membrane subunit	68.2%
b2486	+	*hyfF*	hydrogenase 4, membrane subunit	61.4%
b2488	+	*hyfH*	hydrogenase 4, Fe-S subunit	65.9%
b2489	+	*hyfI*	hydrogenase 4, Fe-S subunit	59.1%
b2490	+	*hyfJ*	predicted processing element hydrogenase 4	68.2%
b2491	+	*hyfR*	DNA-binding transcriptional activator, formate sensing	63.6%
b2492	+	*focB*	predicted formate transporter	65.9%
b2537	-	*hcaR*	DNA-binding transcriptional activator of 3-phenylpropionic acid catabolism	68.2%
b2538	+	*hcaE*	3-phenylpropionate dioxygenase, large (alpha) subunit	63.6%
b2539	+	*hcaF*	3-phenylpropionate dioxygenase, small (beta) subunit	65.9%
b2540	+	*hcaC*	3-phenylpropionate dioxygenase, predicted ferredoxin subunit	68.2%
b2541	+	*hcaB*	2,3-dihydroxy-2,3-dihydrophenylpropionate dehydrogenase	68.2%
b2542	+	*hcaD*	phenylpropionate dioxygenase, ferredoxin reductase subunit	65.9%
b2622	+	*intA*	CP4-57 prophage; integrase	2.3%
b2623	-	*yfjH*	CP4-57 prophage; predicted protein	2.3%
b2624	+	*alpA*	CP4-57 prophage; DNA-binding transcriptional activator	2.3%
b2625	+	*yfjI*	CP4-57 prophage; predicted protein	2.3%
b2626	+	*yfjJ*	CP4-57 prophage; predicted protein	4.5%
b2627	-	*yfjK*	CP4-57 prophage; conserved protein	2.3%
b2628	-	*yfjL*	CP4-57 prophage; predicted protein	2.3%
b2629	-	*yfjM*	CP4-57 prophage; predicted protein	2.3%
b2630	+	*rnlA*	CP4-57 prophage; RNase LS	2.3%
b2631	+	*yfjO*	CP4-57 prophage; predicted protein	2.3%
b2632	+	*yfjP*	CP4-57 prophage; predicted GTP-binding protein	2.3%
b2634	+	*yfjR*	CP4-57 prophage; predicted DNA-binding transcriptional regulator	4.5%
b2635	+	*ypjK*	CP4-57 prophage; predicted inner membrane protein	2.3%

b2636	+	*yfjS*	CP4-57 prophage; predicted protein	4.5%
b2637	+	*yfjT*	CP4-57 prophage; predicted protein	2.3%
b2642	+	*yfjW*	CP4-57 prophage; predicted inner membrane protein	2.3%
b2643	+	*yfjX*	CP4-57 prophage; predicted antirestriction protein	2.3%
b2644	+	*yfjY*	CP4-57 prophage; predicted DNA repair protein	4.5%
b4548	+	*ypjJ*	predicted protein	2.3%
b2645	+	*yfjZ*	CP4-57 prophage; antitoxin of the YpjF-YfjZ toxin-antitoxin system	2.3%
b2646	+	*ypjF*	CP4-57 prophage; toxin of the YpjF-YfjZ toxin-antitoxin system	2.3%
b2665	-	*ygaU*	predicted protein	100.0%
b2714	-	*ascG*	DNA-binding transcriptional repressor	86.4%
b2715	+	*ascF*	fused cellobiose/arbutin/salicin-specific PTS enzymes: IIB component/IC component	84.1%
b2716	+	*ascB*	cryptic 6-phospho-beta-glucosidase	86.4%
b2756	-	*ygcH*	predicted protein	11.4%
b2757	-	*ygcI*	predicted protein	11.4%
b2758	-	*ygcJ*	predicted protein	11.4%
b2759	-	*ygcK*	predicted protein	11.4%
b2760	-	*ygcL*	predicted protein	11.4%
b2761	-	*ygcB*	conserved protein, member of DEAD box family	9.1%
b2782	-	*chpA*	toxin of the ChpA-ChpR toxin-antitoxin system, endoribonuclease	86.4%
b2783	-	*chpR*	antitoxin of the ChpA-ChpR toxin-antitoxin system	84.1%
b2787	-	*gudD*	(D)-glucarate dehydratase 1	100.0%
b2788	-	*gudX*	predicted glucarate dehydratase	86.4%
b2789	-	*gudP*	predicted D-glucarate transporter	100.0%
b2842	-	*kduD*	2-deoxy-D-gluconate 3-dehydrogenase	100.0%
b2843	-	*kduI*	predicted 5-keto 4-deoxyuronate isomerase	100.0%
b2848	+	*yqeJ*	predicted protein	61.4%
b2849	-	*yqeK*	predicted protein	40.9%
b2852	+	*ygeH*	predictedtranscriptional regulator	59.1%
b2853	+	*ygeI*	predicted protein	40.9%
b2902	-	*ygfF*	predicted NAD(P)-binding oxidoreductase with NAD(P)-binding Rossmann-fold domain	40.9%
b3002	-	*yqhA*	conserved inner membrane protein	97.7%
b3003	+	*yghA*	predicted glutathionylspermidine synthase, with NAD(P)-binding Rossmann-fold domain	100.0%
b3021	-	*ygiT*	predicted DNA-binding transcriptional regulator	22.7%
b3022	-	*mqsR*	predicted cyanide hydratase	20.5%
b3027	-	*ygiZ*	conserved inner membrane protein	54.5%
b3043	+	*ygiL*	predicted fimbrial-like adhesin protein	43.2%
b3047	+	*yqiH*	predicted periplasmic pilin chaperone	43.2%
b3048	+	*yqiI*	conserved protein	38.6%
b3072	-	*aer*	fused signal transducer for aerotaxis sensory component/methyl accepting chemotaxis component	97.7%
b3104	+	*yhaI*	predicted inner membrane protein	31.8%
b3119	+	*tdcR*	DNA-binding transcriptional activator	93.2%
b3120	+	*yhaB*	predicted protein	68.2%
b3121	+	*yhaC*	predicted protein	36.4%
b3142	+	*yraH*	predicted fimbrial-like adhesin protein	56.8%
b3143	+	*yraI*	predicted periplasmic pilin chaperone	54.5%
b3144	+	*yraJ*	predicted outer membrane protein	52.3%
b3145	+	*yraK*	predicted fimbrial-like adhesin protein	43.2%
b3151	-	*yraQ*	predicted permease	97.7%
b3153	+	*yhbO*	predicted intracellular protease	88.6%
b3173	-	*yhbX*	predicted hydrolase, inner membrane	95.5%
b3214	+	*gltF*	periplasmic protein	4.5%
b3215	+	*yhcA*	predicted periplasmic chaperone protein	4.5%
b3216	+	*yhcD*	predicted outer membrane protein	4.5%
b3219	+	*yhcF*	predicted transcriptional regulator	4.5%
b3227	+	*dcuD*	predicted transporter	52.3%
b3269	+	*yhdX*	predicted amino-acid transporter subunit	97.7%
b3270	+	*yhdY*	predicted amino-acid transporter subunit	95.5%
b3271	+	*yhdZ*	predicted amino-acid transporter subunit	100.0%
b3322	-	*gspB*	part of gsp divergon involved in type II protein secretion	43.2%
b3323	-	*gspA*	general secretory pathway component, cryptic	38.6%
b3324	+	*gspC*	general secretory pathway component, cryptic	45.5%
b3325	+	*gspD*	general secretory pathway component, cryptic	45.5%
b3326	+	*gspE*	general secretory pathway component, cryptic	45.5%
b3327	+	*gspF*	general secretory pathway component, cryptic	43.2%
b3328	+	*gspG*	pseudopilin, cryptic, general secretion pathway	45.5%
b3330	+	*gspI*	general secretory pathway component, cryptic	43.2%
b3331	+	*gspJ*	predicted general secretory pathway component, cryptic	45.5%
b3332	+	*gspK*	general secretory pathway component, cryptic	40.9%
b3333	+	*gspL*	general secretory pathway component, cryptic	43.2%
b3335	+	*gspO*	bifunctional prepilin leader peptidase/ methylase	36.4%
b3338	-	*chiA*	periplasmic endochitinase	45.5%
b3370	+	*frlA*	predicted fructoselysine transporter	59.1%
b3371	+	*frlB*	fructoselysine-6-P-deglycase	59.1%
b4474	+	*frlC*	predicted isomerase	59.1%

b3374	+	*frlD*	fructoselysine 6-kinase	43.2%
b3375	+	*frlR*	predicted DNA-binding transcriptional regulator	59.1%
b3402	-	*yhgE*	predicted inner membrane protein	100.0%
b3442	+	*yhhZ*	conserved protein	59.1%
b3444	+	*insA*	KpLE2 phage-like element; IS1 repressor protein InsA	27.3%
b3445	+	*insB*	IS1 transposase InsAB'	75.0%
b4612	+	*yrhD*	hypothetical protein	9.1%
b3446	+	*yrhB*	predicted protein	65.9%
b3448	+	*yhhA*	conserved protein	100.0%
b3483	+	*yhhH*	predicted protein	29.5%
b3488	-	*yhiJ*	predicted protein	54.5%
b3501	+	*arsR*	DNA-binding transcriptional repressor	63.6%
b3502	+	*arsB*	arsenite/antimonite transporter	68.2%
b3503	+	*arsC*	arsenate reductase	100.0%
b3505	-	*insH*	IS5 transposase and trans-activator	27.3%
b3508	-	*yhiD*	predicted Mg(2+) transport ATPase inner membrane protein	100.0%
b3524	-	*yhjG*	predicted outer membrane biogenesis protein	100.0%
b3557	+	*insJ*	IS150 protein InsA	22.7%
b3558	+	*insK*	IS150 conserved protein InsB	22.7%
b3562	-	*yiaA*	conserved inner membrane protein	97.7%
b3564	-	*xylB*	xylulokinase	97.7%
b3565	-	*xylA*	D-xylose isomerase	97.7%
b3566	+	*xylF*	D-xylose transporter subunit	97.7%
b3567	+	*xylG*	fused D-xylose transporter subunits of ABC superfamily: ATP-binding components	97.7%
b3568	+	*xylH*	D-xylose transporter subunit	100.0%
b3569	+	*xylR*	DNA-binding transcriptional activator, xylose-binding	97.7%
b3584	-	*yiaT*	predicted protein	63.6%
b3585	+	*yiaU*	predicted DNA-binding transcriptional regulator	65.9%
b3594	+	*yibA*	lyase containing HEAT-repeat	36.4%
b3618	-	*htrL*	predicted protein	45.5%
b3622	+	*rfaL*	O-antigen ligase	4.5%
b3623	-	*waaU*	lipopolysaccharide core biosynthesis	4.5%
b3629	-	*rfaS*	lipopolysaccharide core biosynthesis protein	6.8%
b3645	+	*dinD*	DNA-damage-inducible protein	11.4%
b3656	-	*yicI*	predicted alpha-glucosidase	97.7%
b3657	-	*yicJ*	predicted transporter	97.7%
b3662	-	*nepI*	predicted transporter	97.7%
b4618	+	*tisB*	lexA-regulated toxic peptide	20.5%
b3696	+	*yidX*	predicted lipoproteinC	59.1%
b4557	+	*yidD*	predicted protein	47.7%
b3716	+	*cbrB*	predicted inner membrane protein	70.5%
b3718	-	*yieK*	predicted 6-phosphogluconolactonase	70.5%
b3719	-	*yieL*	predicted xylanase	65.9%
b3720	-	*bglH*	carbohydrate-specific outer membrane porin, cryptic	70.5%
b3721	-	*bglB*	cryptic phospho-beta-glucosidase B	72.7%
b3722	-	*bglF*	fused beta-glucoside-specific PTS enzymes: IIA component/IIB component/IIC component	68.2%
b3723	-	*bglG*	transcriptional antiterminator of the bgl operon	63.6%
b3824	-	*rhtB*	neutral amino-acid efflux system	97.7%
b3861	+	*yihF*	conserved protein	97.7%
b3889	+	*yiiE*	predicted transcriptional regulator	88.6%
b3890	+	*yiiF*	conserved protein	40.9%
b3897	-	*frvR*	predicted regulator	68.2%
b3898	-	*frvX*	predicted endo-1,4-beta-glucanase	68.2%
b3899	-	*frvB*	fused predicted PTS enzymes: IIB component/IIC component	68.2%
b3900	-	*frvA*	predicted enzyme IIA component of PTS	68.2%
b3901	-	*rhaM*	L-rhamnose mutarotase	97.7%
b3902	-	*rhaD*	rhamnulose-1-phosphate aldolase	100.0%
b3903	-	*rhaA*	L-rhamnose isomerase	100.0%
b3904	-	*rhaB*	rhamnulokinase	97.7%
b3905	+	*rhaS*	DNA-binding transcriptional activator, L-rhamnose-binding	97.7%
b3906	+	*rhaR*	DNA-binding transcriptional activator, L-rhamnose-binding	97.7%
b3907	-	*rhaT*	L-rhamnose:proton symporter	90.9%
b3909	+	*kdgT*	2-keto-3-deoxy-D-gluconate transporter	97.7%
b3989	+	*yjaZ*	heat shock protein	36.4%
b4011	+	*yjaA*	conserved protein	38.6%
b4017	-	*arpA*	ankyrin repeat protein, function unknown	20.5%
b4026	+	*yjbE*	predicted protein	68.2%
b4027	+	*yjbF*	predicted lipoprotein	100.0%
b4028	+	*yjbG*	conserved protein	100.0%
b4029	+	*yjbH*	predicted porin	100.0%
b4620	-	*yjbT*	hypothetical protein, no homologs	50.0%
b4031	-	*xylE*	D-xylose transporter	56.8%
b4047	+	*yjbL*	predicted protein	22.7%
b4048	+	*yjbM*	predicted protein	18.2%

b4621	-	*yjbS*	hypothetical protein	2.3%
b4066	-	*yjcF*	conserved protein	47.7%
b4084	-	*alsK*	D-allose kinase	56.8%
b4085	-	*alsE*	allulose-6-phosphate 3-epimerase	56.8%
b4086	-	*alsC*	D-allose transporter subunit	45.5%
b4087	-	*alsA*	fused D-allose transporter subunits of ABC superfamily: ATP-binding components	54.5%
b4088	-	*alsB*	D-allose transporter subunit	56.8%
b4089	-	*rpiR*	DNA-binding transcriptional repressor	56.8%
b4090	+	*rpiB*	ribose 5-phosphate isomerase B/allose 6-phosphate isomerase	56.8%
b4092	-	*phnP*	carbon-phosphorus lyase complex accessory protein	100.0%
b4093	-	*phnO*	predicted acyltransferase with acyl-CoA N-acyltransferase domain	97.7%
b4094	-	*phnN*	ribose 1,5-bisphosphokinase	97.7%
b4095	-	*phnM*	carbon-phosphorus lyase complex subunit	100.0%
b4096	-	*phnL*	carbon-phosphorus lyase complex subunit	100.0%
b4097	-	*phnK*	carbon-phosphorus lyase complex subunit	100.0%
b4098	-	*phnJ*	carbon-phosphorus lyase complex subunit	95.5%
b4099	-	*phnI*	carbon-phosphorus lyase complex subunit	97.7%
b4100	-	*phnH*	carbon-phosphorus lyase complex subunit	100.0%
b4101	-	*phnG*	carbon-phosphorus lyase complex subunit	100.0%
b4102	-	*phnF*	predicted DNA-binding transcriptional regulator of phosphonate uptake and biodegradation	100.0%
b4105	-	*phnD*	phosphonate/organophosphate ester transporter subunit	100.0%
b4106	-	*phnC*	phosphonate/organophosphate ester transporter subunit	97.7%
b4109	+	*yjdA*	conserved protein with nucleoside triphosphate hydrolase domain	97.7%
b4110	+	*yjcZ*	conserved protein	100.0%
b4118	-	*melR*	DNA-binding transcriptional dual regulator	100.0%
b4119	+	*melA*	alpha-galactosidase, NAD(P)-binding	100.0%
b4120	+	*melB*	melibiose:sodium symporter	65.9%
b4128	+	*yjdK*	predicted protein	86.4%
b4204	-	*yjfZ*	predicted protein	47.7%
b4211	-	*ytfG*	NAD(P)H:quinone oxidoreductase	100.0%
b4212	+	*ytfH*	predicted transcriptional regulator	100.0%
b4215	+	*ytfI*	predicted protein	20.5%
b4224	+	*chpS*	antitoxin of the ChpB-ChpS toxin-antitoxin system	59.1%
b4225	+	*chpB*	toxin of the ChpB-ChpS toxin-antitoxin system	59.1%
b4248	-	*yjgH*	predicted mRNA endoribonuclease	68.2%
b4249	-	*yjgI*	predicted oxidoreductase with NAD(P)-binding Rossmann-fold domain	70.5%
b4251	+	*yjgJ*	predicted transcriptional regulator	63.6%
b4253	+	*yjgL*	predicted protein	2.3%
b4257	+	*yjgN*	conserved inner membrane protein	61.4%
b4264	-	*idnR*	DNA-binding transcriptional repressor, 5-gluconate-binding	54.5%
b4265	-	*idnT*	L-idonate and D-gluconate transporter	52.3%
b4266	-	*idnO*	5-keto-D-gluconate-5-reductase	54.5%
b4267	-	*idnD*	L-idonate 5-dehydrogenase, NAD-binding	54.5%
b4268	+	*idnK*	D-gluconate kinase, thermosensitive	52.3%
b4272	+	*insC*	KpLE2 phage-like element; IS2 insertion element repressor InsA	50.0%
b4273	+	*insD*	KpLE2 phage-like element; IS2 insertion element transposase InsAB'	50.0%
b4277	+	*yjgZ*	KpLE2 phage-like element; predicted protein	6.8%
b4278	-	*insG*	KpLE2 phage-like element; IS4 predicted transposase	18.2%
b4279	+	*yjhB*	KpLE2 phage-like element; predicted transporter	13.6%
b4280	+	*yjhC*	KpLE2 phage-like element; predicted oxidoreductase	11.4%
b4655	+	*ythA*	expressed protein	4.5%
b4284	-	*insI*	KpLE2 phage-like element; IS30 transposase	50.0%
b4287	-	*fecE*	KpLE2 phage-like element; iron-dicitrate transporter subunit	40.9%
b4288	-	*fecD*	KpLE2 phage-like element; iron-dicitrate transporter subunit	43.2%
b4289	-	*fecC*	KpLE2 phage-like element; iron-dicitrate transporter subunit	45.5%
b4290	-	*fecB*	KpLE2 phage-like element; iron-dicitrate transporter subunit	45.5%
b4291	-	*fecA*	KpLE2 phage-like element; ferric citrate outer membrane transporter	45.5%
b4292	-	*fecR*	KpLE2 phage-like element; transmembrane signal transducer for ferric citrate transport	45.5%
b4293	-	*fecI*	KpLE2 phage-like element; RNA polymerase, sigma 19 factor	45.5%
b4294	+	*insA*	KpLE2 phage-like element; IS1 repressor protein InsA	52.3%
b4295	-	*yjhU*	KpLE2 phage-like element; predicted DNA-binding transcriptional regulator	13.6%
b4296	-	*yjhF*	KpLE2 phage-like element; predicted transporter	13.6%
b4297	-	*yjhG*	KpLE2 phage-like element; predicted dehydratase	13.6%
b4298	-	*yjhH*	KpLE2 phage-like element; predicted lyase/synthase	13.6%
b4299	-	*yjhI*	KpLE2 phage-like element; predicted DNA-binding transcriptional regulator	13.6%
b4300	-	*sgcR*	KpLE2 phage-like element; predicted DNA-binding transcriptional regulator	13.6%
b4301	-	*sgcE*	KpLE2 phage-like element; predicted epimerase	13.6%
b4302	-	*sgcA*	KpLE2 phage-like element; predicted phosphotransferase enzyme IIA component	13.6%
b4303	-	*sgcQ*	KpLE2 phage-like element; predicted nucleoside triphosphatase	13.6%
b4304	-	*sgcC*	KpLE2 phage-like element; predicted phosphotransferase enzyme IIC component	13.6%
b4565	-	*sgcB*	predicted enzyme IIB component of PTS	13.6%
b4305	-	*sgcX*	KpLE2 phage-like element; predicted endoglucanase with Zn-dependent exopeptidase domain	13.6%
b4306	-	*yjhP*	KpLE2 phage-like element; predicted methyltransferase	15.9%
b4307	-	*yjhQ*	KpLE2 phage-like element; predicted acetyltransferase	15.9%

b4566	-	*yjhX*	conserved protein	15.9%
b4309	-	*yjhS*	conserved protein	81.8%
b4311	-	*nanC*	N-acetylnuraminic acid outer membrane channel protein	84.1%
b4325	-	*yjiC*	predicted protein	65.9%
b4327	-	*yjiE*	predicted DNA-binding transcriptional regulator	86.4%
b4331	+	*kptA*	2'-phosphotransferase	65.9%
b4332	-	*yjiJ*	predicted inner membrane protein	54.5%
b4333	-	*yjiK*	conserved protein	75.0%
b4337	-	*mdtM*	multidrug efflux system protein	70.5%
b4340	-	*yjiR*	fused predicted DNA-binding transcriptional regulator/predicted aminotransferase	59.1%
b4341	+	*yjiS*	conserved protein	52.3%
b4345	-	*mcrC*	5-methylcytosine-specific restriction enzyme McrBC, subunit McrC	4.5%
b4346	-	*mcrB*	5-methylcytosine-specific restriction enzyme McrBC, subunit McrB	4.5%
b4348	-	*hsdS*	specificity determinant for hsdM and hsdR	4.5%
b4351	+	*mrr*	methylated adenine and cytosine restriction protein	13.6%
b4355	+	*tsr*	methyl-accepting chemotaxis protein I, serine sensor receptor	97.7%
b4385	+	*yjjJ*	predicted DNA-binding transcriptional regulator	29.5%

The 680 of K-12 genes that are absent in all of the three completely sequenced *E. albertii* strains are listed. Among these, 88 genes that are conserved in more than 36 strains out of the 44 fully sequenced *E. coli* strains (>80%) are indicated in blue or red. The genes indicated in red are conserved in all 44 *E. coli* strains and absent in all 34 *E. albertii* strains. Gene clusters discussed in the main text were highlighted.
